# Azidocoumarin Glycan Probes for Photoinduced Cross-Linking and In Situ Fluorescent Labeling

**DOI:** 10.1021/acs.bioconjchem.5c00613

**Published:** 2026-03-07

**Authors:** Nina Jahnke, Marc D. Driessen, Georgia Partalidou, Simon Przetak, Ulla I.M. Gerling-Driessen, Laura Hartmann

**Affiliations:** † Department of Organic and Macromolecular Chemistry, 9170 Heinrich Heine University Duesseldorf, Universitaetsstrasse 1, 40225 Duesseldorf, Germany; ‡ Faculty of Medicine and University Hospital Cologne, Department of Oral, Maxillofacial and Plastic Surgery, 14309 University of Cologne, Kerpener Str. 62, 50937 Cologne, Germany; § Institute for Macromolecular Chemistry, 9174 Albert Ludwig University of Freiburg, Stefan-Meier-Strasse 31, 79104 Freiburg, Germany; ∥ Freiburg Center of Interactive Materials and Bioinspired Technologies (FIT), Georges-Köhler-Allee 105, 79110 Freiburg, Germany; ⊥ Freiburg Materials Research Center (FMF), Stefan-Meier-Straße 21, 79104 Freiburg, Germany

## Abstract

Photoinduced affinity labeling for cross-linking biomolecules in close spatial proximity has become a powerful strategy in life science studies to identify interaction partners in fundamental research as well as biomarkers in applied studies. Next-generation photo-cross-linkers additionally provide inducible fluorogenic properties to enable a visual read-out. Azido-substituted coumarin is nonfluorescent, but UV irradiation initiates the formation of a highly reactive nitrene radical that can act as a cross-linker while restoring the fluorescence activity of the coumarin chromophore. In this study, we present a 7-azidocoumarin derivative that is used as a suitable building block for solid-phase synthesis and demonstrates easy access to a variety of glycan-based photo affinity probes. Applications of photo-cross-linkers for glycans and their respective binding proteins are still rare. We show several azidocoumarin glycan-presenting probes and their selective targeting and covalent linking to lectins, accompanied by a turn-on fluorescence activity of the coumarin fluorophore. Selective recognition of specific target lectins from the presented glycan photo affinity probes is further demonstrated in complex biological environments, which now open opportunities for identifying and localizing both known and previously unidentified glycan receptors in cells, tissues, or patient samples.

## Graphical abstract

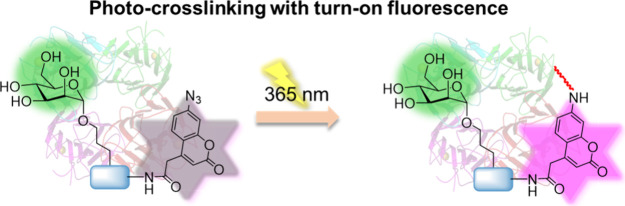

## Introduction

Protein cross-linking is a powerful strategy to study interaction partners of proteins, such as other proteins, glycans, or small molecules. By forming a covalent bond between the interacting partners, this technique enables the capture of transient or even weak associations and thereby allows mapping of the interactome of a target protein, the identification of binding sites, or to gain insights into modes of action.^1,2^

Especially cross-linkers that can be activated upon irradiation with light have been advantageous in such applications due to their spatial and temporal precision. This method is commonly referred to as photo affinity labeling (PAL).^3,4^ One central goal of PAL experiments is to detect and characterize interaction partners of small molecule ligands, such as carbohydrates or peptides.^5^ To this end, various photo affinity probes have been established over the years that are often equipped with aryl azides, diazirenes, or benzophenone derivatives undergoing covalent cross-linking upon irradiation.^6–11^ While alkyl and simple phenyl azides require shorter wavelengths (<250 nm) for activation, aryl azides can be activated by irradiation at higher wavelengths (254–400 nm), leading to the release of N_2_ and the formation of reactive nitrene intermediates.^12,13^ Benzophenone, diazirenes, or substituted aryl azide derivatives are typically photo activated at wavelengths around 350–365 nm, which makes them more suitable for applications in biological samples.^14,15^ The generated nitrenes are then able to initiate various covalent linkage reactions, e.g., to insert into C–H and N–H bonds or result in ring expansion and react with amines as nucleophiles.^16^ Subsequent detection of the covalently bound interaction partners is usually a combination of different enrichment strategies, molecular biology assays, and mass spectrometry.^17–23^

However, a disadvantage of using the above-mentioned derivatives for PAL is a lack of direct visual control of successful cross-linking during the experiment. Having parallel prompting of fluorescence induced by the cross-linking event can offer a great advantage to PAL applications, in particular when targeting low-affinity, multivalent interactions, such as glycan-lectin recognition.^24^ Fluorophores with turn-on properties have been developed for various applications, including live cell imaging,^25,26^ enzyme activity monitoring,^27,28^ biosensing and diagnostic in tumorigenic environments,^29–31^ as well as monitoring in drug delivery applications.^32^ Such fluorogenic probes have also been used in PAL applications, often in combination with RNA or peptide ligands.^33–38^ Surprisingly, fluorogenic PAL probes are still underrepresented for the detection of glycan interactions.^39,40^ Glycan interactions are typically weak (μM to mM range) and thus generally more challenging to detect. In addition, glycan-protein binding is often formed by multivalent glycan ligands that can undergo statistical rebinding in the recognition site of the interacting protein. As a consequence, capturing these short-lived interactions with PAL remains difficult. Fluorogenic detection of successful cross-linking would benefit the detection of glycan interactions; however, fluorogenic glycan-based PAL probes are not easily accessible.

An interesting natural class of fluorophores with fluorogenic properties comprises derivatives of coumarins, which belong to the class of benzopyrones and occur in various plants, such as tonka beans, sweet clover, and cinnamon.^41^ Coumarins have been applied as active agents against diseases like cancer and various infectious diseases.^42,43^ However, due to their variable optical activity, coumarin derivatives are most commonly applied as fluorophores.^44^ The variability in fluorescence emission wavelength depends predominantly on the type of substituent and the substituted position on the coumarin ring.^45^ Especially, the 3- or 4-position of the vinyl group and the 6- or 7-position of the phenyl group are attractive substitution positions and chemically accessible for functionalization.^46,47^ The fluorescence variability ranges across a broad spectrum of emission wavelengths, extending from blue emission in 7-aminocoumarin derivatives,^48,49^ such as 7-amino-4-methylcoumarin (λ_max,Em_ = 435 nm),^50^ over green emission for 7-diethylamino-4-trifluoromethylcoumarin (λ_max,Em_ = 509 nm),^51^ to the near-infrared region through manipulating the π-system of coumarin-based dyes, in which the carbonyl group of the lactone function is replaced by cyano­(4-pyridine/pyrimidine)­methylene moieties (so-called COUPY dyes, λ_max,Em_ > 600 nm).^52,53^

A particularly interesting derivative is azidocoumarin (AzC), which carries an electron-rich azide substituent, typically at position 3 or 7. By attaching the azide to the coumarin backbone, the fluorescence is initially quenched but can be regained by reduction of the azide to a primary amine.^54,55^ Such azide-containing coumarins can react with alkynes as counterparts in a copper-catalyzed azide–alkyne cycloaddition (CuAAC), which also restores fluorescence after reaction.^56^ Godula et al., for instance, attached AzC to glycosaminoglycans (GAGs) to enable strain-promoted azide–alkyne cycloaddition (SPAAC) and covalent attachment to GAG-binding proteins.^57^ The regained fluorescence upon successful coupling allowed real-time monitoring of surface modification. In another study by Chalansonnet et al., an AzC derivative was used to detect the reductive activity of microorganisms and living cells based on the release of H_2_S, which reduces the azide to a primary amine and induces fluorescence of the coumarin.^58^ A recent study by Bousch et al. reported the use of a fluorinated AzC that was conjugated to a fucose moiety.^39^ The final 5,6,8-trifluorinated-7-azido-coumarin probe was generated in a multistep synthesis starting from methyl pentafluoro benzoate and was successfully shown to cross-link to BambL, the fucose-binding lectin of *Burkholderia*. To the best of our knowledge, this is also the first study showing a fluorogenic glycan-based PAL probe. In a follow-up study, Vreulz et al. introduced a trifunctional scaffold for the synthesis of glycan PAL probes.^40^ The scaffold features an *N*-alkoxy-amine that allows the ligation of native oligosaccharides, while other functional groups, such as photo-cross-linkers and reporter tags, can be orthogonally conjugated via amine and carboxylic acid motifs.

In this study, we present a modular synthetic approach based on solid-phase synthesis (SPS) to gain straightforward synthetic access to a variety of fluorogenic glycan-based PAL probes. To this end, we establish a 7-azidocoumarin derivative as a building block for use in SPS and introduce a modular protocol allowing simple variations of glycan motifs (Figure 1A). SPS is a well-established methodology using the stepwise assembly of building blocks to get access to monodisperse, sequence-defined macromolecules—including biomacromolecules such as peptides, oligonucleotides, oligosaccharides, as well as non-natural macromolecules and polymers.^59^ In our previous work, we established the so-called solid-phase polymer synthesis of oligo­(amidoamines) (OAAs) and glyco-OAAs as multivalent glycan mimetics. The combination of established peptide coupling chemistry and tailor-made building blocks allows the synthesis of various glyco-OAAs including different glycan motifs, topologies, valencies, and conjugations, e.g., to lipids to derive amphiphilic glyco-OAAs.^60–62^ Based on this toolbox, our approach uses the new AzC building block in combination with tailor-made building blocks and glycan ligands to derive AzC fluorogenic glycan-based PAL probes. Fast and easy access to a variety of affinity probes is demonstrated by the first set presenting mannose (Man) and galactose (Gal) as different glycan motifs. We then use these AzC probes as double-faceted molecules in PAL applications by establishing their cross-linking abilities combined with distinctive fluorogenic properties by targeting Man- as well as Gal-specific lectins (Figure 1B). We show that fluorescence of the AzC glycan PAL probes is selectively activated only upon cross-linking with the respective target lectin. Furthermore, our study includes the applicability of the AzC glycan PAL probes developed here under different pH conditions, as well as in complex biological environments and cell-based assays, which further enhances the versatility and applicability of PAL in glycan research.

**Figure 1:**
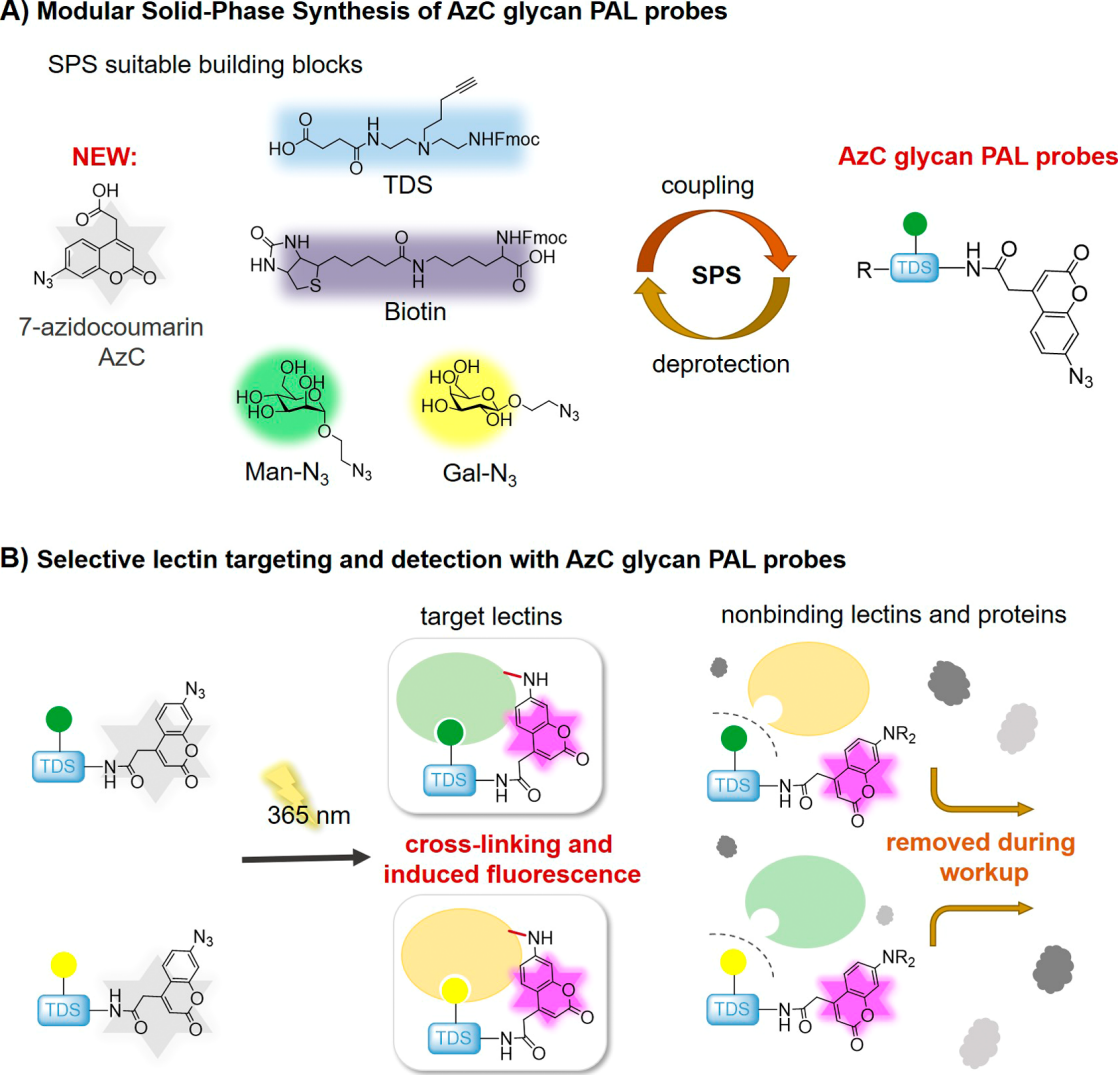
Schematic overview of the study. (A) Use of the AzC building block in SPS to give easy access to novel fluorogenic glycan-based affinity probes for applications in PAL. (B) Schematic concept of selective lectin targeting of Man- and Gal-based AzC affinity probes. Photoactivated probes cross-link their targets, while unbound, activated probes are removed during workup procedures.

## Results

### Synthesis of the 7-Azidocoumarin Building Block and Solid-Phase Synthesis of AzC Glycan PAL Probes

In order to use SPS for straightforward and modular access to a variety of glycan probes, we first synthesized a suitable AzC building block. In addition to the azide moiety, the building block must provide a carboxylic acid function to allow amide coupling following standard solid-phase peptide chemistry. In a one-pot reaction, the primary amine of the commercially available precursor 2-(7-amino-2-oxo-2H-chromen-4-yl)­acetic acid is transferred into an azide group (Figure 2A). The final AzC building block was isolated in high purity, as confirmed by ^1^H NMR and RP-HPCL-MS (see SI, Figures S1–S4).

**Figure 2:**
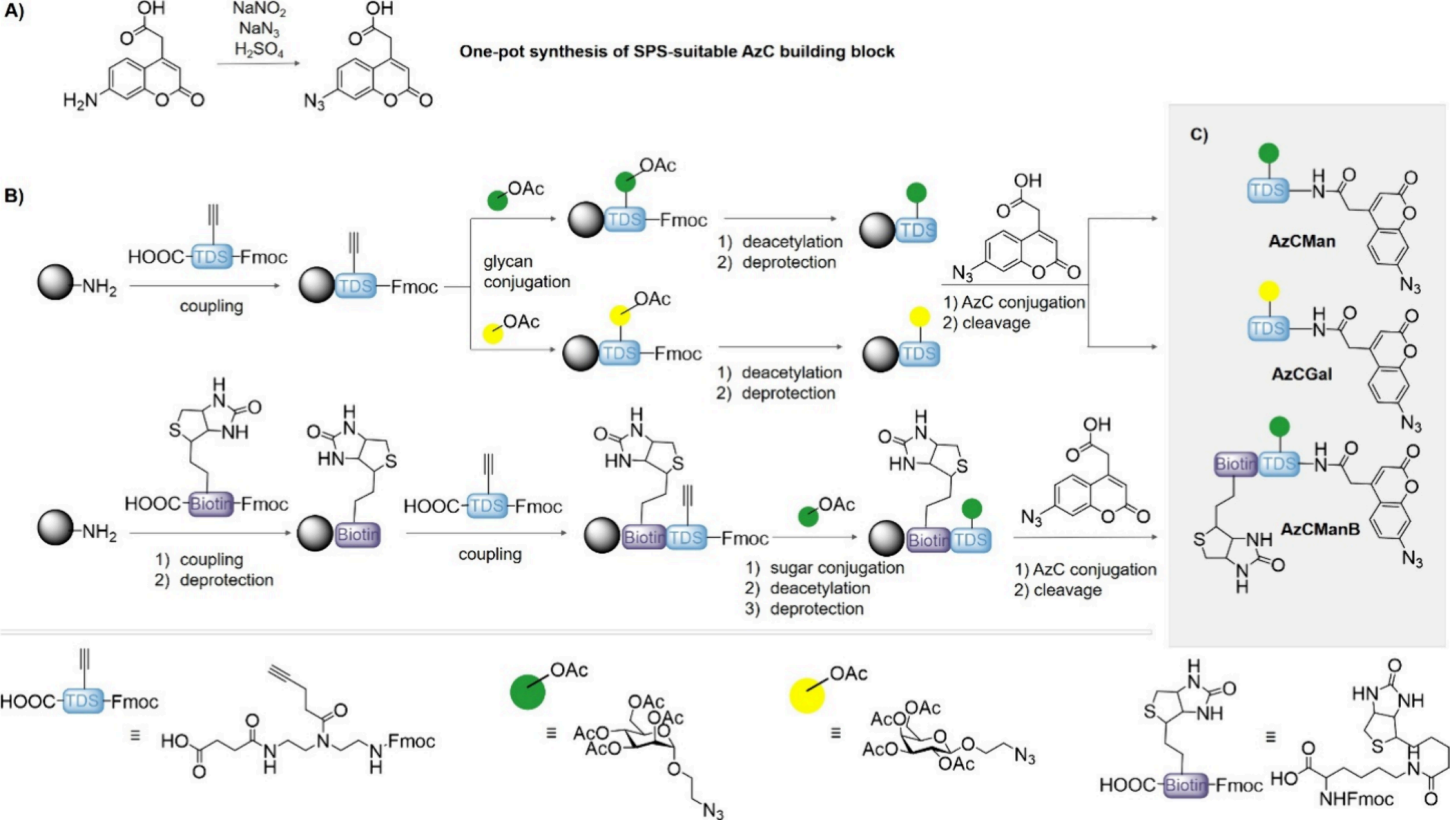
(A) Synthesis of the AzC building block from the aminocoumarin precursor, (B) SPS strategy, and (C) chemical structure of the AzC glycan PAL probes. Synthesis conditions: coupling: 5 eq. building block, 5 eq. PyBOP, 10 eq. DIPEA in DMF for 1 h; deprotection: 25Vol% piperidine in DMF, twice for 10 min, once for 20 min; glycan conjugation: 2.5 eq. Man-N_3_ (or Gal-N_3_) in DMF, 1.25 eq. sodium ascorbate and 1.25 eq. copper sulfate in MiliQ overnight; deacetylation: 0.2 M sodium methanolate in methanol for 1 h; AzC conjugation: 5 eq. AzC building block, 5 eq. HOBt, 5 eq. DIC in DMF for 1 h; resin cleavage: 95Vol% TFA, 2.5Vol% TIPS and 2.5Vol% DCM for 1 h.

Prior to use in SPS, the fluorescence of the AzC building block was evaluated in a 1:1 mixture of acetonitrile and water and compared with the commercially available precursor aminocoumarin. Notably, AzC itself exhibited no discernible extinction within the spectral range of 320–400 nm, which aligns well with prior expectations (see SI, Figure S21A).^63^ To determine the optimal irradiation time that provides a fluorescent signal of the AzC building block, AzC was irradiated for 2–60 min at a wavelength of 365 nm, followed by measuring the fluorescence intensity between 414 and 514 nm (SI, Figure S21B). After 2 min of irradiation, we observed a slight increase in fluorescence, which further increased upon longer irradiation times and reached a maximum at an irradiation duration of 30 min. Structural changes of the AzC building block were analyzed via RP-HPLC. In accordance with the increase in fluorescence over longer irradiation times, we observed a decreasing UV signal of the AzC building block along with the formation of degradation products in the respective RP-HPLC (see SI, Figure S22). Under the used conditions (1:1 acetonitrile/water) several reactions of AzC, such as oxidation, activation, and cycloadditions leading to byproducts with different fluorescence properties,^64^ are possible, which likely explains the decreased fluorescence of AzC after photoactivation in comparison to nonirradiated aminocoumarin.

In order to synthesize the AzC glycan PAL probes, the AzC building block is combined with the previously established building blocks triple-bond diethylenetriamine coupled with succinic acid (TDS), as well as Man-N_3_ and Gal-N_3_, using standard Fmoc-peptide coupling protocols (Figure 2B).^65,66^ All AzC glycan PAL probes consist of one TDS building block, which is conjugated on the alkyne side chain with either Man or Gal via CuAAC. The AzC is coupled in the final step to the N-terminus of the TDS-Man/Gal oligomer. Specifically, an Fmoc-protected TentaGel S-RAM resin is used, to which TDS was coupled as the first building block using benzotriazol-1-yloxytrispyrrolidinophosphonium hexaphosphate (PyBOP) and *N,N*-diisopropylethylamine (DIPEA) as coupling reagents.^67^ Then, acetyl-protected Man- or Gal-N_3_ was conjugated to the alkyne function of TDS using CuAAC. Before N-terminal AzC coupling was performed, the acetyl-protected hydroxy groups of the carbohydrates were deprotected with sodium methanolate, as strong basic pH can result in destruction of the AzC building block. Subsequently, the Fmoc group of TDS was removed, and the AzC building block was coupled using 1-hydroxybenzotriazole (HOBt)/*N,N*-diisopropylcarbodiimide (DIC). The final AzC carbohydrate probe was cleaved off the solid phase using trifluoroacetic acid (TFA) and triisopropylsilane (TIPS). The AzCMan and AzCGal probes (Figure 2C) were isolated with relative purities of >95%, as determined by RP-HPLC (see SI, Figures S5–S14).

Based on the modularity of the solid-phase approach, other functional groups can also be introduced. Here, we synthesized an additional AzC glycan PAL probe containing a biotin motif, which allows for enrichment of the cross-linked biomolecules. For the biotin-containing probe AzCManB, Fmoc-Lys­(biotin)–OH was coupled as the initial building block to the TentaGel S-RAM resin, followed by the same protocol used for AzCMan synthesis. All probes were characterized using RP-HPLC-MS, HR-ESI, IR, and ^1^H NMR (see SI, Figures S15–S20).

### Selective Cross-Linking of AzC Glycan PAL Probes to Target Lectins

Having established a straightforward synthesis strategy suitable for making diverse glycan PAL probes, we used AzCMan and AzCGal to test for specific cross-linking upon binding to selected carbohydrate-recognizing target lectins. Man and Gal were selected as glycan motifs as they represent abundant components in cell surface glycans and are thus involved in a variety of native carbohydrate-lectin interactions.^68,69^ They present an ideal model system for investigating our AzC glycan PAL probe regarding selective interaction and photoinduced cross-linking with the lectin Concanavalin A (ConA), which recognizes Man, even with low affinity, but does not bind to Gal.^70^ *Ricinus communis* agglutinin (RCA_120_), in turn, was used as a Gal-recognizing lectin with no affinity for Man.^71–74^ Therefore, the AzCGal probe is not supposed to be recognized by ConA and serves as a control for evaluating cross-linking selectivity.

Aryl azides are generally able to form very reactive nitrenes by irradiation at 365 nm.^75^ Due to the short-lived, radical character of these nitrenes, covalent binding can only take place when a target protein/lectin is in close spatial proximity to the AzC probe, which is only given for direct interaction partners (i.e., glycan-lectin binding in this case). Therefore, it was expected that only AzCMan, but not the AzCGal probe, forms a covalent bond to ConA upon irradiation (Figure 3A). To confirm this, in a first cross-linking experiment, equimolar amounts (10 μM) of either AzCMan and AzCGal were incubated with ConA for 20 min, followed by irradiation at 365 nm for 15 min, followed by analysis via MALDI-TOF-MS.

**Figure 3:**
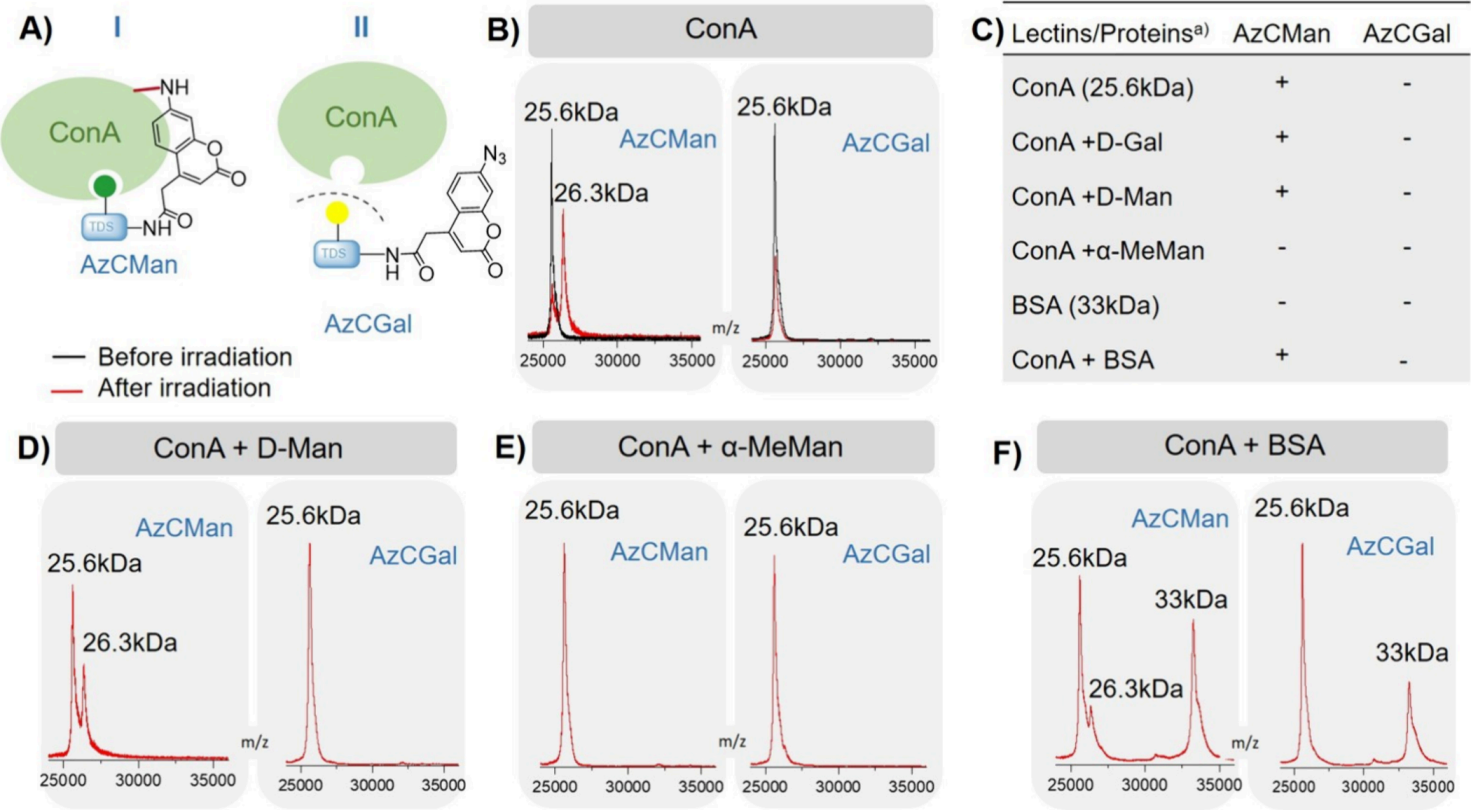
(A) Schematic presentation of ConA binding to AzCMan and nonbinding of the AzCGal probe. (B) MALDI-TOF-MS spectra of AzC glycan PAL probes with ConA before (black) and after (red) irradiation. (C) Overview of results from MALDI-TOF-MS spectra with (+) indicating a peak for ConA cross-linked to AzCMan and (−) reflecting that no peak for ConA-AzCMan or ConA-AzCGal conjugate was found. (D–F) MALDI-TOF-MS spectra of AzC glycan PAL probes with ConA in the presence of competing carbohydrates or proteins: (D) in the presence of D-Man, (E) in the presence of α-MeMan, or (F) in the presence of BSA.

Figure 3B displays the corresponding MALDI-TOF-MS spectra (see SI for full spectra, Figures S23–S26) of the AzC glycan PAL probes with ConA before (black) and after (red) irradiation. As expected, before irradiation, both probes show a single peak corresponding to the molar mass of the ConA monomer (about 25.6 kDa). After irradiation, only the sample incubated with AzCMan displayed an additional peak corresponding to the molecular weight of ConA cross-linked to the AzCMan probe (26.3 kDa). This peak is absent in the sample incubated with AzCGal, suggesting that close spatial proximity to ConA leading to UV-induced cross-linking was only given for AzCMan, but not for the AzCGal probe. It can be assumed that this was the result of the AzCMan probe being bound in the recognition site of ConA. The fact that the AzCGal probe did not show any cross-linking to ConA indicates that the captured interaction was the result of ligand binding rather than nonspecific cross-linking.

To further investigate the selective targeting of the probes, we performed additional PAL experiments with the AzC glycan PAL probes in the presence of competing binding and nonbinding carbohydrates, as well as bovine serum albumin (BSA) as a nonglycan-binding protein (Figures S27–S34). To this end, AzCMan and AzCGal were incubated with ConA and supplemented with a 40-fold excess of D-galactose (D-Gal), D-mannose (D-Man), α-methylmannose (α-MeMan), and BSA. ConA was incubated with the respective competing glycans (D-Gal, D-Man, α-MeMan) or BSA for a duration of 20 min before the AzCMan or AzCGal probes were added in an equimolar ratio to ConA. After addition of the AzC glycan PAL probes, the samples were incubated for another 20 min to allow interaction between the probes and ConA. Subsequently, the samples were irradiated for 15 min at 365 nm and analyzed by MALDI-TOF-MS. Figure 3C shows in the presence of which additional glycans or proteins AzCMan showed successful cross-linking to ConA. The ConA-AzCMan conjugate was detected in the presence of both excess nonbinding Gal and nonbinding BSA, confirming that cross-linking is only achieved from specific glycan-protein interactions. When adding a high excess of binding α-MeMan, as expected, the monosaccharide outcompetes AzCMan and no cross-linking product is observed. Interestingly, supplementing ConA with an excess of D-Man did not prevent cross-linking to the AzCMan probe (Figure 3D). ConA can recognize both D-Man and α-MeMan. Thus, we assumed that both would inhibit efficient binding of the AzCMan probe to ConA. The fact that the D-Man-supplemented sample showed the ConA-AzCMan conjugate despite the competing sugar indicates that the interaction of AzCMan with ConA is stronger compared to ConA with D-Man. In contrast, α-MeMan might form a stronger interaction with ConA compared to AzCMan. This is supported by the much higher binding affinity of α-MeMan to ConA compared to D-Man.^76^ Having a monovalent carbohydrate in the AzCMan probe, it is not unlikely that the binding pocket of ConA was occupied by α-MeMan, preventing additional binding of the AzCMan probe. This result further supports the fact that the AzCMan probe needs to form a tight interaction with the target lectin to form a covalent bond upon irradiation. In addition, incubation with BSA instead of ConA, which should not recognize either of the two carbohydrate moieties as it mainly interacts with hydrophobic molecules,^77,78^ showed no peak corresponding to a potential BSA probe conjugate (see SI, Figures S31 and S32).

Next, a mixture of ConA and BSA was used to assess whether the AzCMan probe can selectively target ConA in the presence of other proteins (Figure 3F). Indeed, in addition to the BSA peak (33 kDa), the ConA-AzCMan conjugate (26.3 kDa) was detected, confirming that the AzCMan probe can selectively cross-link the target protein even in the presence of other nonbinding proteins. Thus, the MALDI-TOF-MS study provided a first indication of the selective cross-linking capabilities of the AzC carbohydrate probes in crowded protein and carbohydrate mixtures.

### Inducible Fluorogenic Properties of AzC Glycan PAL Probes

After establishing successful and selective cross-linking of the AzCMan probe to ConA by MALDI-TOF-MS, we next investigated whether the probe conjugation results in a turn-on fluorescence of the coumarin fluorophore. To this end, we measured the fluorescence of the AzC glycan PAL probes that were incubated with different proteins before and after irradiation with UV light. We used a mixture of ConA and BSA, where ConA serves as the binding protein to AzCMan and BSA as a nonbinding protein to both probes. Likewise, we used a mixture of RCA_120_ and BSA for the AzCGal probe. The AzC probes were incubated for 20 min in equimolar concentrations with the protein mixture and then irradiated for 15 min at 365 nm. Afterward, a purification step was required to isolate the formed lectin-probe conjugates from any unbound AzC probe that might have been activated during irradiation, as these free fluorescent probes could potentially interfere with the fluorescence read-out of the probe-lectin conjugates. Therefore, the irradiated samples were subjected to a centrifugal concentrator with a molecular weight cutoff (MWCO) of 1000 Da to isolate the probe-lectin conjugates. The collected samples were lyophilized and subsequently resuspended in 200 μL lectin-binding buffer (LBB) to maintain the original concentration of 10 μM. Then, the fluorescence of the samples was measured at 450 nm. (Figure 4A,B). In addition, the samples were separated by SDS–PAGE and analyzed using a fluorescent read-out, followed by Coomassie staining of the gel (Figures 4C,D and S37).

**Figure 4:**
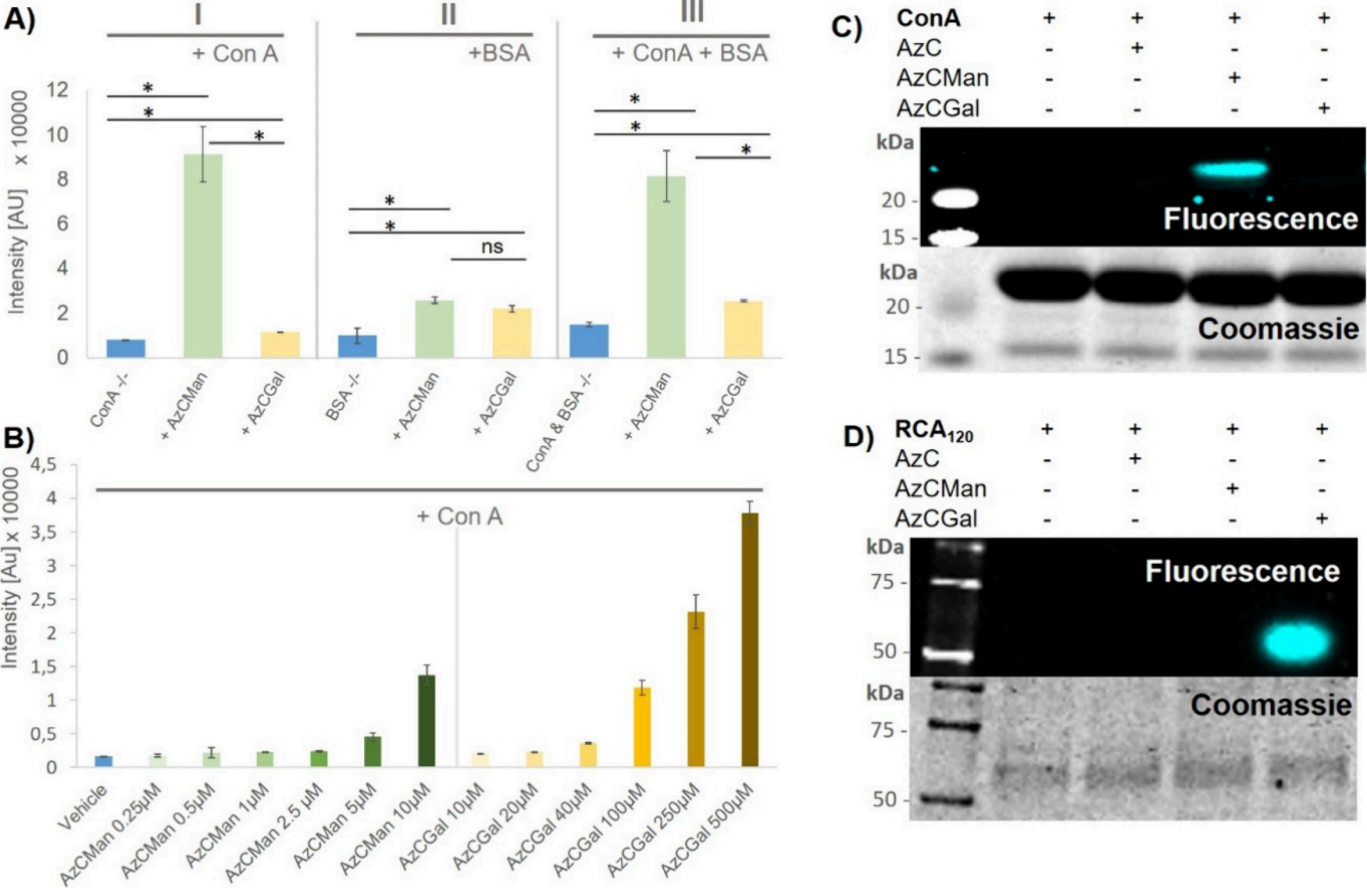
(A) Maximal fluorescence intensities of AzCMan and AzCGal probes at 10 μM concentration incubated with either ConA or BSA alone or a mixture of both proteins. Data represent the mean of four individual measurements. (B) Limit of detection for AzCMan and limit of selectivity for AzCGal. Data represent the mean of four individual measurements. (C) SDS–PAGE of ConA incubated with AzC glycan PAL probes after irradiation at 365 nm; the top panel shows AzC fluorescence and the lower panel shows Coomassie stain of the same gel. (D) SDS–PAGE of RCA_120_ incubated with AzC glycan PAL probes after irradiation at 365nm; the top panel shows AzC fluorescence and the lower panel shows Coomassie stain of the same gel. The complete SDS–PAGE images including the AzCManB probe are shown in the SI (Figure S37).

Figure 4A shows the maximal fluorescence intensities at 450 nm of the different proteins incubated with the AzC glycan PAL probes after irradiation (see SI for detailed fluorescence spectra; Figure S35B). Figure 4A–I corresponds to the probes (AzCMan or AzCGal) incubated with ConA, where a significant increase in fluorescence intensity was only observed with the AzCMan probe, but not with the AzCGal probe. Figure 4A-**II** shows nonbinding BSA incubated with AzCMan or AzCGal, where a marginal increase in fluorescence is observed for both probes, which was attributed to potentially incomplete removal of the free, irradiated probe during the purification step. However, it is essential to note that the fluorescence intensity resulting from the interaction with BSA is considerably lower compared with the ConA samples. Figure 4A-**III** shows the fluorescence intensities of the samples where the two AzC glycan PAL probes were incubated with a mixture of ConA and BSA. Here, the difference in fluorescence intensity is clearly visible. The sample containing the AzCMan probe shows a significant increase in fluorescence activity in contrast to the sample containing the AzCGal probe. These results align well with the MALDI-TOF analysis and provide further evidence that the AzCMan probe can be selectively cross-linked to its target lectin ConA, and that this is accompanied by inducible fluorescence of the coumarin unit.

We next evaluated whether the probes are prone to nonspecific photoinduced cross-linking at high concentrations. In addition, we were interested in determining the minimal probe concentration at which a photoinduced turn-on fluorescence could still be detected for probe-target cross-linking. Thus, on the one hand, we explored the limit of detection (LOD) at low probe concentration and, on the other hand, studied potential nonspecific cross-linking at high probe concentrations. To this end, we incubated ConA with increasing concentrations of both AzCMan and AzCGal and measured the maximum fluorescence intensities at 450 nm (Figure 4B). For AzCMan, concentrations between 0.25 and 10 μM were selected, where we observed an increase in fluorescence intensities with increasing probe concentrations. Between 0 and 5 μM, the fluorescence intensities increased in an almost linear fashion (see SI, Figure S35C,D), which was used to calculate an LOD of 2.4 μM (see SI, Figure S35E) for AzCMan interacting with ConA. This value indicates a high sensitivity of the AzCMan probe for detecting an interaction with ConA. Carbohydrate-lectin interactions are usually of very low affinity, especially for monovalent glycans. For comparison, the binding constant of α-MeMan to ConA has a *K*_d_ of 130 μM.^79^ The comparable low LOD of AzCMan shows that even low-affinity interactions can be successfully detected with this AzC glycan PAL probe. To test for nonspecific binding of AzCGal to ConA, the concentration of the probe was increased up to 500 μM while keeping the ConA concentration at 10 μM, resulting in a 50-fold excess of probe relative to protein. As shown in Figure 4B, the fluorescence intensity increases notably at probe concentrations higher than 100 μM. We attribute this to nonspecific binding at large probe excess, as Gal is well-known not to show any affinity to ConA and is therefore used as the nonbinding control in studies investigating Man-ConA interactions.^80–82^ However, using the probes at an equimolar or up to a 5-fold higher concentration in relation to the target protein ensures specific target interaction with no detectable nonspecific binding.

Finally, we validated the applicability of the AzC glycan PAL probes in biochemical assays. Using SDS–PAGE, we tested whether the fluorescence of the cross-linked conjugates (ConA-AzCMan and RCA_120_-AzCGal) could be detected in-gel (Figure 4C,D). Here, the AzC building block not containing any glycan motifs was included as a control to exclude nonspecific binding of the AzC moiety itself. In Figure 4C, the upper panel displays the AzC fluorescence image of the gel. After fluorescence measurement, the gels were stained with Coomassie as a control for sample loading across the lanes. The Coomassie staining of the gels is displayed in the lower panel. For both lectins, a fluorescent band only occurs when they are incubated with the respective binding glycan probe during irradiation (AzCMan in the case of ConA and AzCGal in the case of RCA_120_). Thus, SDS–PAGE analysis confirmed the selective target cross-linking combined with induced fluorescence for the AzC glycan PAL probes.

### Stability of the Induced AzC Probe Fluorescence at Different pH Ranges

Considering that biological applications can be accompanied by dynamic pH fluctuations, it is crucial to evaluate whether the photoinduced fluorescence of the cross-linked species is stable in acidic or basic environments. Thus, next, we tested the stability of the induced coumarin fluorescence at various pH conditions. Ramesh et al. demonstrated that the lactone ring of coumarin can undergo ring opening under basic conditions.^83^ This could potentially lead to a shift or quenching of the fluorescence. In order to cover a wide pH range, we measured the fluorescence of the cross-linked probes at pH 4–10. AzCMan was incubated with ConA and irradiated as previously described. The unbound AzC glycan PAL probe was removed. The isolated and lyophilized AzCMan-ConA conjugates were then resuspended, and the individual probe solutions were adjusted to pH 4, 5, 6, 7, and 8 to give a final probe concentration of 5 μM. The fluorescent measurements of the samples were carried out after an incubation period of 60 min at the individual pH values. All samples showed strong fluorescence at 450 nm, independent of the pH range (see SI, Figure S35A). The individual fluorescence spectra show marginal variations in absolute intensities, which can be attributed to standard fluctuations during fluorescence measurements and minimal variations in the sample concentration rather than being an effect of the individual pH value. Thus, we conclude that the photoinduced fluorescence of cross-linked AzC glycan PAL probe conjugates is stable over a wide pH range.

### Evaluation of the Minimal Irradiation Time to Gain Stable Probe-Target Cross-Linking

Based on these previous results, next, we established the optimal irradiation times that are required to gain successful and robust cross-linking of the probe to its target protein. Therefore, time-dependent irradiation experiments were carried out by mixing ConA and the AzCMan probe in equimolar ratios (20 μM) and incubating the samples for 20 min before irradiation between 2 and 60 min at 365 nm with a fixed energy input control (0.33 J/cm^2^/min). The irradiated samples were subsequently separated by SDS–PAGE, and the fluorescence intensities of the AzCMan-ConA conjugates were quantified relative to the Coomassie stain intensities of the same lanes (Figure 5A,B).

**Figure 5:**
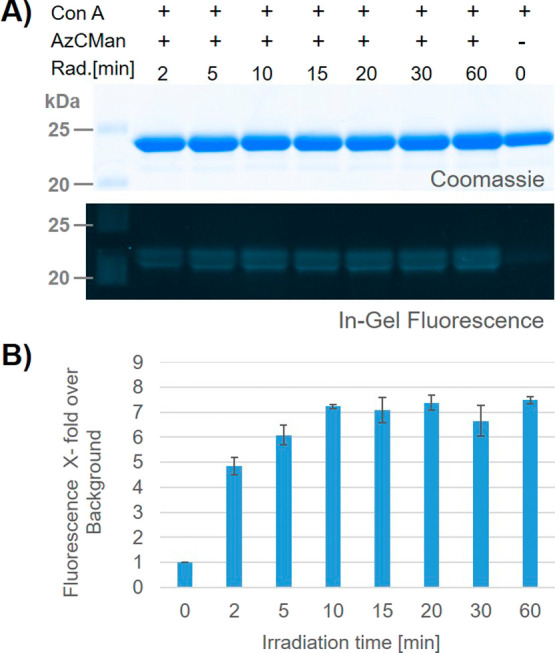
(A) Coomassie-stained and fluorescence image of the SDS–PAGE containing ConA with AzCMan at different durations of irradiation; the top panel shows Coomassie stain; the lower panel shows AzC fluorescence of the same gel. (B) Quantification of the fluorescence intensities of the individual bands normalized against ConA (last lane on the gel). The quantification was calculated as the mean of two replicates of the time-dependent irradiation series applied on one SDS–PAGE (full image provided in the SI, Figure S36).

Figure 5A shows the images of the Coomassie stain (top) and the fluorescence read-out (bottom) of the same gel. The bands represent AzCMan conjugated to ConA at irradiation durations of 2, 5, 10, 15, 20, 30, and 60 min. The last lane contains Con A without the AzCMan probe and serves as a reference (see SI for full-size image of the gel showing two replicates, Figure S36). For all irradiation durations, a fluorescence band was detected in th gel. The fluorescence intensities differ only marginally for the individual irradiation durations. Notably, a fluorescent band can already be detected after irradiation of ConA in the presence of AzCMan for only 2 min, indicating rapid AzCMan cross-linking to ConA. Figure 5B shows the quantified fluorescence intensities of the individual bands normalized to the ConA band. The fluorescence intensity peaks around the 10 min mark and does not significantly increase further with longer irradiation times. It is worth noting that even for long irradiation times of up to 60 min, no significant bleaching of the fluorophore or dissociation of the probe was observed. Hence, robust photoinduced cross-linking can be achieved for the AzCMan probe within 2 min of irradiation at 365 nm. This offers opportunities for expedited analysis suitable for unstable or sensitive samples or for rapid screening approaches. Since the induced fluorescence is not quenched during long irradiation durations, the probe also seems suitable for long-term irradiation applications. Hence, the duration of irradiation can be individually adapted to the respective demands of the experiment without the risk of losing cross-linking efficiency or fluorescence intensity.

### Selective Target Recognition in Complex Biological Environments

Biological systems, such as cell lysates, intact cells, or organelles, are complex mixtures of different proteins, lipids, peptides, and glycans. To be used in molecular biology and biochemical assays, the AzC glycan PAL probes and the cross-linked conjugates need to be structurally stable under these conditions. In addition, selective and immediate cross-linking to the target protein upon photoactivation is desired. Thus, AzC glycan PAL probes were tested in complex mixtures derived from cells and animal organs, again looking at the selective cross-linking to their respective target proteins upon photoactivation without showing random fluorescence activation due to probe discomposure or nonspecific cross-linking to other components in the mixture.

In the first experiment, AzC glycan PAL probes were incubated with cell lysate generated from MDA-MB 231 cells. The proteins in the cell lysate were denatured using SDS. The probes were incubated either with the cell lysate alone or with cell lysate supplemented with ConA. ConA is a plant-based lectin; it is not present in human cell lines, such as MDA-MB321 cells. In this test, we also included the more complex probe containing Man and biotin (AzCManB) to show whether the more complex probe would still bind the target lectin (ConA) as well, which would, in a next step, offer isolation of the cross-linked target via the biotin handle. AzCGal and AzC without any carbohydrate ligand served as controls for nonspecific binding to other proteins in the lysate. We incubated 20 μL of cell lysate (2 mg/mL) with 5 μL of vehicle or ConA (50 μM) and 10 μL of probe (50 μM) and incubated these mixtures for 20 min, followed by UV irradiation for 15 min. Subsequently, the samples were applied to SDS–PAGE. AzC fluorescence was detected in the gel, followed by Coomassie staining (Figure 6A). The fluorescence read-out of the gel shows fluorescent bands only in samples where ConA was supplemented to the lysate and only for probes containing the Man residue (AzCMan and AzCManB). Interestingly, the fluorescence intensity was higher in the case of incubation of ConA with AzCManB compared to AzCMan. This could indicate that the biotin present in this probe could increase the binding affinity to ConA. Since lectin binding is a low-affinity on–off integration, especially for monosaccharide ligands, we speculate that the sterically more demanding AzCManB probe undergoes less rapid statistical rebinding in the carbohydrate recognition site of ConA, leading to a higher ratio of the cross-linked probe to the lectin. However, a more detailed analysis of this effect will be subject to a detailed kinetic comparison of the probes in a follow-up study. The test in cell lysate revealed that even in complex protein mixtures, we do not observe nonspecific cross-linking of the AzC glycan PAL probes. To test whether the biotin in the AzCManB probe is still accessible after cross-linking to the target, we used streptavidin binding. We incubated the AzC building block and all AzC glycan PAL probes with ConA. After irradiation, we applied all samples to SDS–PAGE and subsequent Western blot (WB). After detecting AzC fluorescence for the bands containing AzCMan and AzCManB (see SI, Figure S38A), we incubated the WB with streptavidin conjugated to DyLight 790 and visualized the binding of streptavidin, which was found exclusively in the band with the biotin-containing AzCManB probe (see SI, Figure S38B). This result confirms that the biotin in the AzCManB probe is still accessible for streptavidin binding after cross-linking to the target protein, thus allowing isolation of the cross-linked conjugates via streptavidin affinity columns.

**Figure 6:**
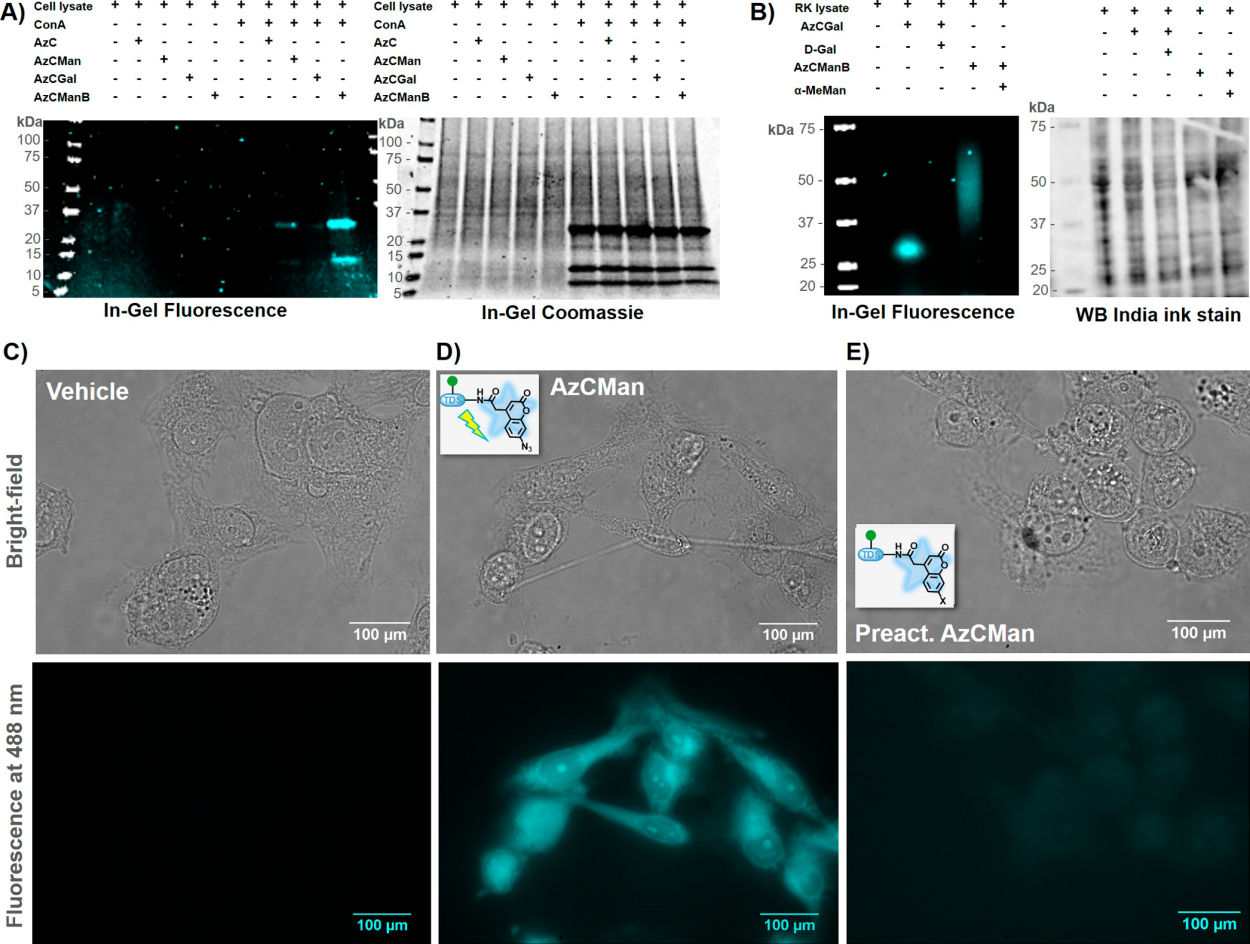
(A) SDS–PAGE showing MDA-MB 231 lysate with or without supplemented ConA that was incubated with various AzC glycan PAL probes and subsequently irradiated with UV light for 15 min. (B) Western blot of native rat kidney lysate that was incubated with AzCGal and AzCManB probes in the presence or absence of the respective carbohydrate inhibitors (α-MeMan and D-Gal) and subsequently irradiated with UV light for 15 min. (C–E) Microscope images (top: bright-field; bottom: fluorescence at 488 nm) of fixed MDA-MB-231 cells incubated with vehicle (C), AzCMan (D), or preactivated AzCMan (E) and subsequently irradiated at 365 nm for 15 min.

Next, we studied whether AzCMan and AzCGal could be used to identify unknown interaction partners in biological settings. In native lysates, the structure and function of the proteins are kept intact. Hence, it is expected that proteins in native lysates contain lectin domains specific for Man or Gal ligands, which should potentially be recognized and cross-linked by the AzC glycan PAL probes upon photoactivation. Successful cross-linking would be detectable by fluorescent bands in the gel. We prepared a native lysate of rat kidneys and incubated it with AzCGal and AzCManB for 20 min, followed by 15 min UV irradiation and SDS–PAGE separation (Figure 6B). Indeed, we detected fluorescent bands in the native lysate gel after incubation with the AzCGal and AzCManB probes independently. This was not surprising as the native rat kidney lysate was likely to contain both Man- and Gal-recognizing receptors. Interestingly, the fluorescent bands appear at different molecular weights for the AzCGal and AzCManB probes, indicating that the two probes cross-linked to different target proteins. This was a first indication that the cross-linked conjugates were the result of a carbohydrate-specific interaction with the respective unidentified protein/lectin targets. To verify this, we also treated the native lysate with the two AzC glycan PAL probes in the presence of an excess of free D-Gal and α-MeMan (5-fold excess compared to the AzC glycan PALprobe). These monosaccharides should act as inhibitors for the two carbohydrate probes, and as a consequence, no fluorescent bands should be detected for the AzC glycan PAL probes in the presence of D-Gal and α-MeMan. Indeed, the rat kidney samples that contained the AzC glycan PAL probes in the presence of the monosaccharide inhibitors did not show the fluorescent bands that were detected for the AzC probes without D-Gal and α-MeMan. Thus, it can be concluded that the fluorescent bands in the native rat kidney lysate originate from cross-linking of the AzC glycan PAL probes to specific Gal- and Man-recognizing receptors. These results underline the usefulness of these or similar AzC carbohydrate probes for the detection and subsequent isolation of specific carbohydrate-interacting proteins.

After having established that the photoinduced fluorescence of the AzC glycan PAL probes can be detected using fluorescence spectroscopy and SDS–PAGE analysis, we now wanted to validate the visualization of the AzC glycan PAL probes using fluorescence microscopy in cell-based assays. To the best of our knowledge, this is the first time showing that AzC glycan PAL probes were tested for application in cell studies. We again used MDA-MB-231 cells, which offer a variety of carbohydrate-recognizing receptors on the cell surface. In these cell studies, we tested only the AzCMan probe. Since there are also Gal-recognizing receptors on the cell surface, no significant distinction could be expected by treating the cells with AzCMan or AzCGal. Since we did not detect nonspecific cross-linking with the unconjugated AzC building block, we used preirradiated AzCMan as a control probe in this setup. To this end, AzCMan was irradiated at 365 nm for 15 min before addition to the cells. Due to prior irradiation, the AzCMan probe becomes fluorescent. Containing the mannose ligand, the preactivated AzCMan can still bind to mannose-recognizing proteins on the cell surface. However, the probe has lost the ability to cross-link and thus covalently bind to interacting targets during the irradiation procedure and is therefore largely removed during the washing procedures. This allows a distinction of the fluorogenic properties of simply target-bound versus covalently linked AzCMan.

Cells were grown in 8-well chamber microscopy slides to 80% confluency and fixed using ice-cold methanol. Subsequently, vehicle control, AzCMan, or deactivated AzCMan was added to the cells. After incubation for 20 min, the plate was irradiated for 15 min at 365 nm. Afterward, the cells were washed thoroughly with PBS to remove the unbound AzCMan probe. The cells were then visualized using a fluorescence microscope in bright-field mode and at a fluorescence excitation of 365 nm (Figure 6C–E; and SI Figures S39–S41). During preirradiation of AzCMan, the azide is removed, which turns on the fluorescence of the AzC probe. However, due to the loss of the azide, this preactivated probe should not be able to cross-link to protein targets on the cells during irradiation and will be removed during the washing steps following the irradiation step. It serves as a control to determine if the detected fluorescence can either be attributed to the cross-linked AzC probe or is the result of receptor-bound, but noncross-linked probe.

All cells are clearly visible in the bright-field images (Figure 6C–E, upper panel). LBB-treated cells (vehicle) show no fluorescence at 488 nm (Figure 6C, lower panel), proving that no autofluorescence of the cells can be detected at this wavelength. Cells treated with AzCMan (Figure 6D) show bright fluorescence at 488 nm, indicating that AzCMan was cross-linked to Man-recognizing proteins. The negative control in which cells were incubated with the preactivated AzCMan probe (Figure 6D) shows only marginal fluorescence at 488 nm, likely resulting from residual probe that remained target-bound even throughout the washing procedure. In comparison to the AzCMan probe that was activated on the cells upon irradiation, a clear difference in fluorescence intensity is evident. These results show that the AzC carbohydrate probes can also be applied in cell-based assays.

Finally, we wanted to explore the possibility if the laser of the microscope could be used to locally induce probe cross-linking. The excitation wavelength of the laser in our instrument is 365 nm, which is in the range to induce photo-cross-linking for azidocoumarin probes. The AzCMan probe was added to MDA-MB231 cells, and an image in bright-field mode was immediately taken. Afterward, we switched to fluorescence emission at 488 nm. Images were taken at several intervals, starting from *t* = 0 to *t* = 10 min (see SI, Figure S42). We observed a steady increase in fluorescence intensities starting after 1 min of laser irradiation. Given that in this setup the unbound probe cannot be washed out, a high background signal is observed in these images. However, this proof-of-concept study showed that cross-linking of AzC glycan PAL probe can be induced locally on cells using the UV laser of the microscope. This offers new potential applications and experimental setups using these probes, such as initiating and life tracking of cross-linking events in cells.

## Conclusions

Taken together, we exploited the double-faceted mode of action of a new 7-azidocoumarin derivative that can simultaneously act as a photo-cross-linker and a fluorogenic turn-on dye. We established an AzC building block suitable for SPS and demonstrated the successful and straightforward synthesis of different glycan-based AzC PAL probes. In contrast to classical organic synthesis, the SPS approach enables easy access and variation of the probes, e.g., with regard to glycan type, architecture, and valency.^84^ Specifically, as a first set, we synthesized and characterized AzCMan and AzCGal probes and investigated their applicability as photo-cross-linking affinity probes in a series of lectin interaction and cell-based assays. The AzC glycan PAL probes showed highly selective photoinduced cross-linking only in the case of direct interaction with the respective target lectin. We confirmed this in studies using additional nonbinding proteins and competing carbohydrates. The photoinduced cross-linking of the AzC glycan PAL probes is accompanied by an induced fluorescence of the coumarin dye, which can be detected in solution, in SDS–PAGE and Western blots, as well as in cell imaging using fluorescence microscopy. We found selective target cross-linking for the AzC glycan PAL probes even in complex environments, such as cell- or whole organ lysates. Finally, we were able to show that under native conditions, the AzC glycan PAL probes have the potential to detect interaction partners specific for the respective glycan motif. In conclusion, the SPS-suitable AzC building block offers high potential for the development of a variety of affinity probes, including multivalent glycan or peptide-based probes, for a broad spectrum of bioimaging applications.

## Methods

### General

No unexpected or unusually high safety hazards were encountered during the synthesis or performance of the experiments.

Acetone (≥99.8%) was purchased from Fischer Scientific. Diethyl ether (with BHT as inhibitor, ≥99.8%), triisopropylsilane (TIPS) (98%), bovine serum albumin ((≥96%, powder) (+)-sodium-L-ascorbate (≥99%), *N*,*N*-diisopropylcarbodiimide (99%), sodium nitrite), deuteriumoxide-d2 (99,8 atom %), sulfuric acid (95.0–97.0%), and 1-hydroxybenzotriazole (≥97%) were purchased from Sigma-Aldrich. *N*,*N*-Diisopropylethylamine (DIPEA) (≥99%) and potassium hydroxide (≥85%) were purchased from Carl Roth. Methanol (100%), D-galactose (≥99%), ethyl acetate (>99.9%), *n*-hexane (≥99.8%), and acetic anhydride (99.7%) were purchased from VWR BDH Prolabo Chemicals. *N*,*N*-Dimethylformamide (DMF) (99.8%, for peptide synthesis), piperidine (99%), sodium methoxide (97%), sodium diethyldithiocarbamate (99%), copper­(II)­sulfate (98%), and sodium azide (≥97%) were purchased from Acros Organics. D-Mannose (≥98%) was purchased from Merck. Dichloromethane (DCM) (99.9%), triethyl silane (≥98%), trifluoroacetic acid (≥99,0%), and benzotriazole-1-yl-oxy-tris-pyrrolidino-phosphonium (PyBOP) were purchased from Iris Biotech GmbH. Methyl-α-D-mannopyranoside (>99%) was purchased from Cytiva. Ethanol (>99.9%) was purchased from Chemsolute. Concanavalin A (highly purified, power) was purchased from MP Biomedicals. Lectin from *Ricinus communis* agglutinin (RCA_120_) was purchased from BIOZOL. The anion resin (AG1-X8, quaternary ammonium, 100–200 mesh, acetate form) was purchased from Bio-Rad. 2-(7-Amino-2-oxo-2H-chromen-4-yl) acetic acid (97%) was purchased from BLDPharm. TentaGel resin was purchased from Rapp Polymere. Water/H_2_O used here is ultra pure water, drawn from a Milli-Q water purification system.

### Nuclear Magnetic Resonance Spectroscopy (NMR)

^1^H NMR and ^13^C NMR spectra were recorded on a Bruker Avance III 300. Chemical shifts were reported as delta (δ) in parts per million (ppm) and coupling constants as J in Hertz (Hz). Multiplicities are stated as follows: s = singlet, d = doublet, t = triplet, q = quartet, m = multiple.

### High-Resolution Mass Spectrometry (HR-MS)

HR-MS measurements were conducted on a Bruker UHR-QTOF maXis 4G with a direct inlet via syringe pump, an ESI source, and a quadrupole time-of-flight (QTOF) analyzer. Samples were dissolved in water at a concentration of 1 mg/mL.

### Matrix-Assisted Laser Desorption Ionization Time-of-Flight (MALDI-TOF) Mass Spectrometry (MALDI-TOF-MS)

MALDI-TOF measurements were conducted on an Ultraflex I instrument from Bruker Daltonics. The samples were measured in linear mode with cyano-4-hydroxycinnamic acid (HCCA) as matrix in a ratio of 1:2. As a solvent, H_2_O/MeCN­(1:1) or lectin-binding buffer (LBB) was used.

### Reversed-Phase High-Pressure Liquid Chromatography (RP-HPLC)

RP-HPLC was performed with an Agilent 1260 Infinity instrument coupled to a variable wavelength detector (VWD) set to 214 nm. As a column, a Poroshell 120 EC-C18 1.8 μM (3.0 × 50 mm, 2.5 μM) reversed-phase column was used. Mobile phase A consisted of 95 vol%/5 vol% H_2_O/MeCN with 0.1 vol% formic acid, and mobile phase B consisted of 95 vol%/5 vol% MeCN/H_2_O with 0.1 vol% formic acid. The flow rate for all measurements was 0.4 mL/min

### Synthesis of Building Blocks for Solid-Phase Synthesis

The building blocks TDS (triple-bond diethylenetriamine succinic acid), Man-N_3_ (tetra-*O*-acetyl-azidoethyl-α-d-mannopyranoside) , and Gal-N_3_ (tetra-*O*-acetyl-azidoethyl-β-d-galactopyranoside) were synthesized following previously published protocols.^65,85,86^

### Synthesis of Azidocoumarin (AzC)

First, 650 mg (2.97 mmol) of 7-amino-4-carboxymethylcoumarin was dissolved in 80 mL of distilled water. The solution was cooled in an ice bath for approximately 20 min. While maintaining the temperature in the ice bath, 20 mL of concentrated sulfuric acid was added dropwise over a period of 10 min using a dropping funnel. In parallel, 308 mg (4.45 mmol) of sodium nitrite was dissolved in 20 mL of ice-cold distilled water. This solution was then added dropwise to the reaction mixture over a period of 10 min. The mixture was stirred for another 10 min on ice. Then, 1.2 g (17.8 mmol) of sodium azide was dissolved in 20 mL of ice-cold distilled water and added dropwise to the reaction mixture over a period of 20 min (caution: foam formation). The reaction mixture was stirred for 12 h at room temperature. Afterward, the reaction mixture was diluted with distilled water and extracted 5 times with 100 mL of ethyl acetate. The combined organic phases were washed with a solution of saturated sodium chloride and dried over sodium sulfate. The solvent was removed under reduced pressure, and the product remained as a yellow solid.

### General Procedure for Solid-Phase Synthesis

AzC glycan PAL probes were synthesized using solid-phase synthesis with the Fmoc-standard protocol. TentaGel S-RAM (0.23 mmol/g) was used as the solid phase. The batch size for each glycooligomer was 0.1 mmol.

### Coupling and Fmoc Deprotection

First the resin was swollen 2 times in DCM for 15 min. The swollen resin was then washed 3 times with DMF. Fmoc deprotection was performed using 5 mL of 25 vol% piperidine in DMF. Deprotection was carried out 3 times (2 x for 10 min, 1 x for 20 min). After deprotection, the resin was washed 10 times with DMF. Then, the TDS building block was coupled to the deprotected amine using a solution of 5 eq. building block, 5 eq. PyBOP, and 10 eq. DIPEA in DMF for 1 h. Following the coupling reaction, the resin was washed 10 times with DMF to remove excess reagents.

### Copper­(I)-Catalyzed Alkyne–Azide Cycloaddition (CuAAC)

Glycoconjugation to the TDS backbone was performed using CuAAC. For this purpose, 2.5 eq. of acetylated Man-N_3_ (or Gal-N_3_) was dissolved in 4 mL of DMF. Separately, 1.25 eq. of sodium ascorbate and 1.25 eq. of copper sulfate were each dissolved in 0.25 mL of ultra pure H_2_O. First, the copper sulfate solution was drawn into a syringe, followed by the carbohydrate solution, and finally the sodium ascorbate solution. The syringe reactor was wrapped in aluminum foil to protect it from light and shaken overnight at room temperature. Following the reaction, the resin was washed extensively with a 23 mM solution of sodium diethyl thiocarbamate in DMF/H_2_O (1:1, v/v) until the washing solution was colorless, indicating that thecopper had been fully removed. Final washes were performed with DCM to ensure complete removal of any remaining reagents.

### Removing Acetyl Protection Groups from Carbohydrates

After successful conjugation, the acetyl groups were removed by incubating the resin in a solution of 0.2 M sodium methanolate in methanol for 1 h. Subsequently, the resin was washed 5 times with methanol, 5 times with DCM, and 5 times with DMF.

### Conjugation of Azidocoumarin

After carbohydrate conjugation and acetyl deprotection, the azidocoumarin building block was coupled to the N-terminus. 5 eq. AzC building block, 5 eq. HOBt, and 5 eq. DIC were dissolved in DMF and coupled for 1 h. The reactor was wrapped in aluminum foil to protect it from light. Subsequently, the resin was washed 10 times with DCM and 10 times with DMF.

### Cleavage from the Resin

After completion of the synthesis, the final structures were cleaved off the resin using a cleavage cocktail consisting of 95 vol% TFA, 2.5 vol% TIPS, and 2.5 vol% DCM. The resin was shaken for 1 h at room temperature. Following cleavage, the reaction mixture was poured into 45 mL of ice-cold diethyl ether. The resulting precipitate was collected by centrifugation, and the supernatant was decanted. The crude product was dried under a gentle stream of nitrogen. Subsequently the solid was dissolved in 5 mL of H_2_O and lyophilized using an Alpha 1–4 LD plus instrument (Martin Christ Freeze-Dryers GmbH) at a pressure of 0.1 mbar.

### Irradiation Experiments

For the general irradiation experiments, equimolar amounts of lectin and AzC glycan PAL probe were mixed in lectin-binding buffer (LBB) (10 mM HEPES, 50 mM NaCl, 1 mM MnCl_2_, 1 mM CaCl_2_, pH 7.4). The total reaction volumes varied between 25 and 200 μL, depending on the specific experimental setup. Prior to irradiation, the samples were incubated for 20 min at room temperature to allow binding of the carbohydrate to the respective target. Irradiation was carried out using either a UV-LED Spot P standard at 365 nm (Opsytec Dr. Gröbel GmbH) or a Vilber Biolink blx-365. For quantifying the fluorescence gain at various irradiation time points to assess the photoreaction kinetics, the energy was set to 1 J/cm^2^ per 3 min.

### General Procedure or Irradiation Experiments

Con A (20 μM, 1 eq.) and AzC probes (20 μM, 1 eq.) were mixed in equal volume (1:1, v/v) and incubated for 20 min prior to irradiation, which was then performed as previously described.

### Competition Assays with Carbohydrates

100 μL of Con A (20 μM, 1 eq.) and 5 μL of carbohydrate (16 mM, 40 eq.) were mixed and incubated for 20 min. Afterward, 100 μL of AzC probe (20 μM, 1 eq.) was added and incubated for further 20 min prior to irradiation, which was then performed as previously described.

### Competition Assays with BSA

50 μL of Con A (20 μM, 0.5 eq.) and 50 μL of BSA (20 μM, 0.5 eq.) were mixed and incubated for 20 min. Afterward, 100 μL of AzC probe (40 μM, 1 eq.) was added and incubated for further 20 min prior to irradiation, which was then performed as previously described.

### ConA- and BSA-Fluorescence Measurements

100 μL of ConA (20 μM) or 100 μL of BSA (20 μM) were mixed with 100 μL of AzC probe and incubated for 20 min following the irradiation process as described. Furthermore, a mixture of 50 μL of ConA (40 μM) and 50 μL of BSA (40 μM) was prepared and incubated for 20 min. Then, 100 μL of AzC probe was added and incubated for further 20 min following the irradiation process as described. Unbound probes were separated using a centrifugal concentrator (MWCO 1000 Da), and the remaining sample was lyophilized. The samples were resuspended in LBB, and fluorescence measurements at 450 nm were performed on a CLARIOstar microplate reader (BMG LABTECH) at ambient temperature. Data were evaluated using BMG Mars software. Quadruplicates of all samples were measured in 384 black-well plates from Greiner BIO-ONE. Welch’s T-test (not assuming equal variance) was performed for maximum robustness. P-values were calculated for each incubation condition compared to the respective vehicle control within each group (I, II, II) of the experiment. Analysis ToolPak was used to perform *t-*test evaluation.

### Absorption and Fluorescence Measurements

AzC was dissolved in acetonitrile/water 1:1 to a final concentration of 2 mM, and UV absorption and fluorescence spectra were measured on a CLARIOstar microplate reader (BMG LABTECH) at ambient temperature prior to irradiation. Subsequently, the sample was irradiated at 365 nm for 1, 10, 15, 30, and 60 min. Fluorescence spectra were measured after each time point, and the samples were analyzed by RP-HPLC to check for photoactivation and decomposition. Nonirradiated aminocoumarin (2 mM in acetonitrile/water 1:1) was used as a control in UV and fluorescence measurements.

### pH-Dependent Fluorescence Measurements

100 μL of ConA (20 μM) was mixed with 100 μL of AzC probe (20 μM) and incubated for 20 min following the irradiation process as described. Unbound probes were separated by using a centrifugal concentrator (MWCO 1000 Da) and then lyophilized. The samples were resuspended in 40 μL of H_2_O and divided into four parts of 10 μL each. 90 μL of LBB solution with pH values adjusted to 7, 8, 9, and 10 was added to each sample. The entire procedure was repeated for the pH values of 4, 5, and 6. All solutions were added to the plate as technical triplicates of 30 μL each. The samples were incubated for 1h before fluorescence was measured at 450 nm on a CLARIOstar microplate reader (BMG LABTECH) at ambient temperature.

### Concentration-Dependent Fluorescence Measurements

100 μL of ConA (20 μM) were mixed with 100 μL of AzCMan (at 20, 10, 5, 2, 1, and 0.5 μM) or 100 μL of AzCGal (20, 40, 80, 200, 500, and 1000 μM) and incubated for a further 20 min following the irradiation process as described. Unbound probes were separated using a centrifugal concentrator (MWCO 1000 Da) and then lyophilized. The samples were resuspended in a final volume of 200 μL of LBB. Fluorescence was measured at 450 nm as technical quadruplicates using a CLARIOstar microplate reader (BMG LABTECH) at ambient temperature.

### Statistical Analysis LOD

The limit of detection was calculated as LOD = 3σ/*S*, where σ represents the standard deviation of the blank measurement and *S* represents the linear scope. The LOD was calculated in a range of 0–5 μM using 6 values.

### General SDS–PAGE Procedure

SDS–PAGE was performed in an electrophoresis chamber. 4–20% Mini-PROTEAN TGX Precast Protein Gels, 12 or 15 wells (Bio-Rad), were used with Tris/glycine/SDS as running buffer. Samples were mixed with 4× Laemmli buffer and then denatured at 75 °C for 10 min prior to application to the gel. Proteins were separated over 35 min at 200 V and 300 mA. Precision Plus Protein Kaleidoscope Prestained Protein Standard (Bio-Rad) or Dual Xtra standard protein ladder (Bio-Rad) was used as molecular weight reference. Gels and Western blots were analyzed using an Amersham ImageQuant 800 (Cytiva). After detecting AzC fluorescence, gels were stained with a total protein Coomassie blue stain and destained until no background was visible. Subsequently, gels were analyzed in the imager OD mode with the following exposure settings: colorimetric, automatic, and binning 1 × 1. The exposure time was determined automatically. Blots were stained with India Ink unless stated otherwise and washed multiple time with H_2_O before imaging.

### Duration of Irradiation Measurements

Con A (15 μL, 20 μM) and AzCMan (15 μL, 20 μM) were mixed and incubated for 20 min. The samples were then irradiated for 2, 5, 10, 15, 20, 30, and 60 min at a wavelength of 365 nm using a Vilber Biolink blx-365 with 1 J/cm^2^ per 3 min and subsequently analyzed by SDS–PAGE. Gels were analyzed for fluorescence activity and subsequently Coomassie-stained. Using Bio-Rad Image lab software, “adjusted volumes” (background-subtracted intensity values) were calculated. All fluorescence intensities were calculated in a rectangle (17.86 mm^2^) around the gel band. The same method was used to generate the respective absorption intensities from the Coomassie-stained gel (using identical rectangles of 17.55 mm^2^). The calculated fluorescence values were divided by the values for Coomassie absorption of the same band to control for loading differences. These resulting values were normalized to the untreated control, which did not contain any probe.

### Selective Binding of AzC Probes to Lectins ConA and RCA_120_

10 μL of each AzC probe (50 μM each) was mixed with 10 μL of either ConA (50 μM) or RCA_120_ (50 μM) in a total volume of 80 μL of LBB. The mixture was incubated at room temperature for 20 min, followed by irradiation at 365 nm in a Vilber Biolink blx-365 for 15 min. The samples were subsequently mixed with 4× Laemmli buffer, and the proteins were denatured at 70 °C for 15 min, followed by separation via SDS–PAGE. After measuring in-gel fluorescence, the gels were stained with Coomassie.

### Selective ConA Binding of AzCMan Probes in Cell Lysate Background

MDA-MB231 whole cell lysate was purchased from Santa Cruz Biotechnology (sc-2232). 20 μL of cell lysate (stock 2.5 mg/mL) was mixed with 5 μL of ConA (5 mg/mL), 10 μL of AzC probe (50 mM stock), and 45 μL of LBB. The mixture was incubated for 20 min, followed by 15 min irradiation at 365 nm using a Vilber Biolink blx-365. Afterward, 4× Laemmli buffer was added to the samples and the proteins were separated by SDS–PAGE. After measuring in-gel fluorescence, the gel was stained with Coomassie.

### Testing AzC Probes in Native Rat Kidney Lysate

Fresh-frozen slices of rat kidney were lysed by two-step homogenization in IP buffer (25 mM Tris–HCl, pH 7.4, 150 mM NaCl, 1 mM EDTA, 1% NP-40) containing protease inhibitors. Slices (176 mg) were lysed in buffer (1.8 mL) and divided into three portions of 600 μL each. Each step contained homogenization/disruption in a bead mill (TissueLyser LT, Qiagen) at 50 Hz for 5 min, using a steel ball in a 2 mL protein low-bind reaction tube, followed by 10 min centrifugation at 15k × g and treatment in a sonication bath for 5 min. After each cycle, the supernatant was removed and collected. All samples were kept on ice for the whole procedure. The lysate was checked via SDS–PAGE and Coomassie staining, and equal concentrations were used for native binding experiments. Animals were originally processed for other experiments, and surplus lysates were used in these procedures. All animals were kept in accordance and with approval of the German animal welfare authorities (reference number HHU/O10/87).

For native binding experiments, the following mixtures were prepared. In a total volume of 70 μL of LBB, 30 μL of native rat kidney lysate (1 mg/mL) was mixed with 4 μL of either AzCGal or AzCMan (500 μM each) in the absence or presence of an excess of the respective carbohydrate inhibitors D-Gal or α-MeMan (20 μL of a 500 μM stock). The mixtures were incubated for 20 min, followed by 15 min of irradiation at 365 nm using a Vilber Biolink blx-365. Afterward, 4× Laemmli buffer was added to the samples, and the proteins were separated by SDS–PAGE. Proteins were transferred to an Immobilon-FL PVDF membrane (pore size: 0.45 μm, Millipore) using a Trans-Blot Turbo Transfer System (Bio-Rad). After measuring fluorescence as detailed above the membrane was stained with India Ink.

### Testing AzC Probes on Cells

MDA-MB-231 cells were purchased from the German Collection of Microorganisms and Cell Cultures GmbH of the Leibniz Institute DSMZ (ACC 732).

Cells were cultured according to standard protocols using RPMI-1640 medium supplemented with 10% fetal calf serum and 1% penicillin/streptomycin. Cells were incubated at 37 °C with 5% CO_2_. For testing AzCMan cross-linking, cells were seeded into μ-Slide 8-well chambers at a density of approximately 20,000 cells per well, followed by incubation at 37 °C and 5% CO_2_ over 2 days to allow attachment of the cells to the slides. The medium was then removed, and cells were washed 3 times with 150 μL of PBS buffer. Cells were fixed by treating them with 100 μL of ice-cold methanol for 3–5 min. Subsequently, cells were washed 3 times with 150 μL of PBS and then covered with 100 μL of PBS. 20 μL of a 60 μM stock solution of AzC probe in LBB was added to give a final concentration of 10 μM. Plates were gently mixed for 2 min and then incubated for 20 min to allow the probe to interact with Man-binding proteins. Then, each well was irradiated successively for 15 min using a UV-LED Spot P standard at 365 nm (Opsytec Dr. Gröbel GmbH) at a power of 100%. Before imaging, each well was washed 2 times with 150 μL of PBS. Fluorescence microscopy was performed on an Olympus IX73 microscope using a 60× oil objective (Gain 300; Exposure 40). For the preparation of the inactivated negative control, 30 μL of AzCMan (60 μM) was irradiated prior to incubation with the cells.

## Supplementary Material



## Notes

Analytical data for the synthesized structures, additional data and figures showing the absorption and fluorescence spectra of AzC building blocks, full MALDI-TOF-MS and full fluorescence spectra, additional fluorescence spectra for the pH-stability study, additional SDS–PAGE images of irradiated and not irradiated lysates, and additional fluorescence microscopy images (PDF)

## References

[1] Jayachandran B., Parvin T. N., Alam M. M., Chanda K., Mm B. (2022). Insights on
Chemical Crosslinking Strategies for Proteins. Molecules.

[2] Liu J., Yang B., Wang L. (2023). Residue selective
crosslinking of
proteins through photoactivatable or proximity-enabled reactivity. Curr. Opin. Chem. Biol..

[3] Kotzyba-Hibert F., Kapfer I., Goeldner M. (1995). Recent trends in photoaffinity
labeling. Angew. Chem., Int. Ed..

[4] Bayley H., Knowles J. R. (1977). Photoaffinity labeling. Methods
Enzymol..

[5] Yu S.-H., Wands A. M., Kohler J. J. (2012). Photoaffinity
probes for studying
carbohydrate biology. J. Carbohydr. Chem..

[6] Dorman G., Nakamura H., Pulsipher A., Prestwich G. D. (2016). The life
of pi star: exploring the exciting and forbidden worlds of the benzophenone
photophore. Chem. Rev..

[7] Galardy R. E., Craig L. C., Jamieson J. D., Printz M. P. (1974). Photoaffinity labeling
of peptide hormone binding sites. J. Biol. Chem..

[8] Fleet G., Porter R., Knowles J. (1969). Affinity labelling
of antibodies
with aryl nitrene as reactive group. Nature.

[9] Kym P. R., Carlson K. E., Katzenellenbogen J. A. (1995). Evaluation
of a highly efficient
aryl azide photoaffinity labeling reagent for the progesterone receptor. Bioconjugate Chem..

[10] Smith R. A., Knowles J. R. (1973). Aryldiazirines. Potential reagents for photolabeling
of biological receptor sites. J. Am. Chem. Soc..

[11] Mishra P. K., Yoo C. M., Hong E., Rhee H. W. (2020). Photo-crosslinking:
an emerging chemical tool for investigating molecular networks in
live cells. ChemBioChem..

[12] Leyva E., Platz M. S., Persy G., Wirz J. (1986). Photochemistry of phenyl
azide: the role of singlet and triplet phenylnitrene as transient
intermediates. J. Am. Chem. Soc..

[13] Walrant A., Sachon E. (2025). Photoaffinity labeling
coupled to MS to identify peptide
biological partners: Secondary reactions, for better or for worse?. Mass Spectrom. Rev..

[14] Murale D. P., Hong S. C., Haque M. M., Lee J. S. (2017). Photo-affinity labeling
(PAL) in chemical proteomics: a handy tool to investigate protein-protein
interactions (PPIs). Proteome Sci..

[15] Liang T. Y., Schuster G. B. (1987). Photochemistry of 3- and 4-nitrophenyl
azide: detection
and characterization of reactive intermediates. J. Am. Chem. Soc..

[16] Wentrup C. (2011). Nitrenes,
carbenes, diradicals, and ylides. Interconversions of reactive intermediates. Acc. Chem. Res..

[17] Chowdhry V., Westheimer F. (1979). Photoaffinity labeling of biological systems. Annu. Rev. Biochem..

[18] Wu H., Kohler J. (2019). Photocrosslinking probes
for capture of carbohydrate
interactions. Curr. Opin. Chem. Biol..

[19] Jahn O., Eckart K., Tezval H., Spiess J. (2004). Characterization of
peptide–protein interactions using photoaffinity labeling and
LC/MS. Anal. Bioanal. Chem..

[20] Robinette D., Neamati N., Tomer K. B., Borchers C. H. (2006). Photoaffinity labeling
combined with mass spectrometric approaches as a tool for structural
proteomics. Expert review of proteomics.

[21] Tulloch L. B., Menzies S. K., Fraser A. L., Gould E. R., King E. F., Zacharova M. K., Florence G. J., Smith T. K. (2017). Photo-affinity labelling
and biochemical analyses identify the target of trypanocidal simplified
natural product analogues. PLoS Neglected Tropical
Diseases.

[22] Smith E., Collins I. (2015). Photoaffinity labeling in target-and
binding-site identification. Future medicinal
chemistry.

[23] Park J., Koh M., Koo J. Y., Lee S., Park S. B. (2016). Investigation of
specific binding proteins to photoaffinity linkers for efficient deconvolution
of target protein. ACS Chem. Biol..

[24] Wagner S., Hauck D., Hoffmann M., Sommer R., Joachim I., Müller R., Imberty A., Varrot A., Titz A. (2017). Covalent Lectin
Inhibition and Application in Bacterial Biofilm Imaging. Angew. Chem., Int. Ed..

[25] Wang Y., Torres-García D., Mostert T. P., Reinalda L., Van Kasteren S. I. (2024). A Bioorthogonal
Dual Fluorogenic Probe for the Live-Cell Monitoring of Nutrient Uptake
by Mammalian Cells. Angew. Chem., Int. Ed..

[26] Yu A., He X., Shen T., Yu X., Mao W., Chi W., Liu X., Wu H. (2025). Design strategies for tetrazine fluorogenic
probes
for bioorthogonal imaging. Chem. Soc. Rev..

[27] Jeong H., Wu X., Lee J.-S., Yoon J. (2023). Recent advances in enzyme-activated
NIR fluorescent probes for biological applications. TrAC Trends in Analytical Chemistry.

[28] Cosco E. D., Bogyo M. (2024). Recent advances in
ratiometric fluorescence imaging of enzyme activity
in vivo. Curr. Opin. Chem. Biol..

[29] Scott J. I., Deng Q., Vendrell M. (2021). Near-infrared
fluorescent probes
for the detection of cancer-associated proteases. ACS Chem. Biol..

[30] Saleem M., Hanif M., Bonne S., Zeeshan M., Khan S., Rafiq M., Tahir T., Lu C., Cai R. (2024). Turn-On Fluorescence
Probe for Cancer-Related γ-Glutamyltranspeptidase Detection. Molecules.

[31] Tsao K. K., Imai S., Chang M., Hario S., Terai T., Campbell R. E. (2024). The best of both
worlds: Chemigenetic fluorescent sensors
for biological imaging. Cell Chemical Biology.

[32] Nadal-Bufi F., Salomon P. L., de Moliner F., Sarris K. A., Wang Z., Wills R. D., Marin V. L., Shi X., Zhou K., Wang Z., Xu Z., McPherson M. J., Marvin C. C., Hobson A. D., Vendrell M. (2025). Fluorogenic Platform
for Real-Time Imaging of Subcellular Payload Release in Antibody–Drug
Conjugates. J. Am. Chem. Soc..

[33] Kellner S., Seidu-Larry S., Burhenne J., Motorin Y., Helm M. (2011). A multifunctional
bioconjugate module for versatile photoaffinity labeling and click
chemistry of RNA. Nucleic Acids Res..

[34] Morimoto S., Tomohiro T., Maruyama N., Hatanaka Y. (2013). Photoaffinity casting
of a coumarin flag for rapid identification of ligand-binding sites
within protein. Chem. Commun..

[35] Tomohiro T., Yamamoto A., Tatsumi Y., Hatanaka Y. (2013). [3-(Trifluoromethyl)-3
H-diazirin-3-yl] coumarin as a carbene-generating photocross-linker
with masked fluorogenic beacon. Chem. Commun..

[36] Singha M., Roy S., Pandey S. D., Bag S. S., Bhattacharya P., Das M., Ghosh A. S., Ray D., Basak A. (2017). Use of azidonaphthalimide
carboxylic acids as fluorescent templates with a built-in photoreactive
group and a flexible linker simplifies protein labeling studies: applications
in selective tagging of HCAII and penicillin binding proteins. Chem. Commun..

[37] Képiró M., Várkuti B. H., Rauscher A. A., Kellermayer M. S., Varga M., Málnási-Csizmadia A. (2015). Molecular
tattoo: subcellular confinement of drug effects. Chemistry & biology.

[38] Dai S.-Y., Yang D. (2020). A visible and near-infrared
light activatable diazocoumarin probe
for fluorogenic protein labeling in living cells. J. Am. Chem. Soc..

[39] Bousch C., Vreulz B., Kansal K., El-Husseini A., Cecioni S. (2023). Fluorogenic Photo-Crosslinking of Glycan-Binding Protein
Recognition Using a Fluorinated Azido-Coumarin Fucoside. Angew. Chem., Int. Ed..

[40] Vreulz B., De Crozals D., Cecioni S. (2025). A trifunctional probe for generation
of fluorogenic glycan-photocrosslinker conjugates. RSC chemical biology.

[41] Brown, S. A. &nbsp;Biochemistry of The Coumarins, in Biochemistry of Plant Phenolics, Swain, T. ; Harbone, J. B. ; Van Sumere, C. F. , Eds., Springer US: Boston, MA, 1979; pp 249–286.

[42] Musa M. A., Cooperwood J. S., Khan M. O. F. (2008). A review of coumarin derivatives
in pharmacotherapy of breast cancer. Curr. Med.
Chem..

[43] Stringlis I. A., de Jonge R., Pieterse C. M. J. (2019). The Age of Coumarins
in Plant–Microbe
Interactions. Plant Cell Physiol..

[44] Sun X.-y., Liu T., Sun J., Wang X.-j. (2020). Synthesis and application of coumarin
fluorescence probes. RSC Adv..

[45] Tasior M., Kim D., Singha S., Krzeszewski M., Ahn K. H., Gryko D. T. (2015). π-Expanded
coumarins: synthesis, optical properties and applications. Journal of Materials Chemistry C.

[46] Szwaczko K. (2022). Coumarins
synthesis and transformation via C–H bond activation—A
review. Inorganics.

[47] Kang D., Ahn K., Hong S. (2018). Site-Selective C–
H Bond Functionalization of
Chromones and Coumarins. Asian Journal of Organic
Chemistry.

[48] Tod M., Prevot M., Poulou M., Farinotti R., Chalom J., Mahuzier G. (1989). Chromatographic
and luminescence
properties of a 7-aminocoumarin derivative with peroxyoxalate chemiexcitation. Anal. Chim. Acta.

[49] Murase T., Yoshihara T., Yamada K., Tobita S. (2013). Fluorescent
peptides
labeled with environment-sensitive 7-aminocoumarins and their interactions
with lipid bilayer membranes and living cells. Bull. Chem. Soc. Jpn..

[50] Nowakowska M., Smoluch M., Sendor D. (2001). The effect of cyclodextrins on the
photochemical stability of 7-amino-4-methylcoumarin in aqueous solution. J. Incl. Phenom. Macrocycl. Chem..

[51] Raju B. B., Costa S. M. (1999). Excited-state behavior
of 7-diethylaminocoumarin dyes
in AOT reversed micelles: size effects. J. Phys.
Chem. B.

[52] Gandioso A., Palau M., Bresolí-Obach R., Galindo A., Rovira A., Bosch M., Nonell S., Marchán V. (2018). High photostability
in nonconventional coumarins with far-red/NIR emission through azetidinyl
substitution. Journal of Organic Chemistry.

[53] Rovira A., Pujals M., Gandioso A., López-Corrales M., Bosch M., Marchán V. (2020). Modulating
photostability and mitochondria
selectivity in far-red/NIR emitting coumarin fluorophores through
replacement of pyridinium by pyrimidinium. Journal
of Organic Chemistry.

[54] Lord S. J., Lee H.-l. D., Samuel R., Weber R., Liu N., Conley N. R., Thompson M. A., Twieg R. J., Moerner W. (2010). Azido push–
pull fluorogens photoactivate to produce bright fluorescent labels. J. Phys. Chem. B.

[55] De-La-Cuesta J., González E., Pomposo J. A. (2017). Advances in fluorescent single-chain
nanoparticles. Molecules.

[56] Sivakumar K., Xie F., Cash B. M., Long S., Barnhill H. N., Wang Q. (2004). A fluorogenic
1, 3-dipolar cycloaddition reaction of 3-azidocoumarins and acetylenes. Org. Lett..

[57] Porell R. N., Follmar J. L., Purcell S. C., Timm B., Laubach L. K., Kozirovskiy D., Thacker B. E., Glass C. A., Gordts P. L. S. M., Godula K. (2022). Biologically Derived Neoproteoglycans for Profiling
Protein–Glycosaminoglycan Interactions. ACS Chem. Biol..

[58] Chalansonnet V., Lowe J., Orenga S., Perry J. D., Robinson S. N., Stanforth S. P., Sykes H. E., Truong T. V. (2019). Fluorogenic
7-azidocoumarin
and 3/4-azidophthalimide derivatives as indicators of reductase activity
in microorganisms. Bioorg. Med. Chem. Lett..

[59] Hill S. A., Gerke C., Hartmann L. (2018). Recent Developments
in Solid-Phase
Strategies towards Synthetic, Sequence-Defined Macromolecules. Chem.—Asian J..

[60] Blawitzki L.-C., Bartels N., Bonda L., Schmidt S., Monzel C., Hartmann L. (2024). Glycomacromolecules to Tailor Crowded and Heteromultivalent
Glycocalyx Mimetics. Biomacromolecules.

[61] Jäck N., Hemming A., Hartmann L. (2024). Synthesis
of Dual-Responsive Amphiphilic
Glycomacromolecules: Controlled Release of Glycan Ligands via pH and
UV Stimuli. Macromol. Rapid Commun..

[62] Hoffmann M., Snyder N. L., Hartmann L. (2022). Glycosaminoglycan Mimetic Precision
Glycomacromolecules with Sequence-Defined Sulfation and Rigidity Patterns. Biomacromolecules.

[63] Pǎunescu E., Louise L., Jean L., Romieu A., Renard P.-Y. (2011). A versatile
access to new halogenated 7-azidocoumarins for photoaffinity labeling:
Synthesis and photophysical properties. Dyes
Pigm..

[64] Yang Q., Váňa J., Klán P. (2022). The complex photochemistry of coumarin-3-carboxylic
acid in acetonitrile and methanol. Photochemical
& Photobiological Sciences.

[65] Ponader D., Wojcik F., Beceren-Braun F., Dernedde J., Hartmann L. (2012). Sequence-defined
glycopolymer segments presenting mannose: synthesis and lectin binding
affinity. Biomacromolecules.

[66] Wojcik F., Lel S., O’Brien A. G., Seeberger P. H., Hartmann L. (2013). Synthesis of homo-and heteromultivalent
carbohydrate-functionalized
oligo (amidoamines) using novel glyco-building blocks. Beilstein journal of organic chemistry.

[67] Ponader D., Maffre P., Aretz J., Pussak D., Ninnemann N. M., Schmidt S., Seeberger P. H., Rademacher C., Nienhaus G. U., Hartmann L. (2014). Carbohydrate-Lectin
Recognition of
Sequence-Defined Heteromultivalent Glycooligomers. J. Am. Chem. Soc..

[68] Cummings R. D. (2022). The mannose
receptor ligands and the macrophage glycome. Curr. Opin. Struct. Biol..

[69] Coombs P. J., Taylor M. E., Drickamer K. (2006). Two categories
of mammalian galactose-binding
receptors distinguished by glycan array profiling. Glycobiology.

[70] Weatherman R. V., Mortell K. H., Chervenak M., Kiessling L. L., Toone E. J. (1996). Specificity of C-glycoside complexation by mannose/glucose
specific lectins. Biochemistry.

[71] Itakura Y., Nakamura-Tsuruta S., Kominami J., Sharon N., Kasai K.-i., Hirabayashi J. (2007). Systematic
Comparison of Oligosaccharide Specificity
of Ricinus communis Agglutinin I and Erythrina Lectins: a Search by
Frontal Affinity Chromatography†. J.
Biochem..

[72] Wang Y., Yu G., Han Z., Yang B., Hu Y., Zhao X., Wu J., Lv Y., Chai W. (2011). Specificities of Ricinus communis
agglutinin 120 interaction with sulfated galactose. FEBS Lett..

[73] Bojar D., Meche L., Meng G., Eng W., Smith D. F., Cummings R. D., Mahal L. K. (2022). A Useful Guide to
Lectin Binding:
Machine-Learning Directed Annotation of 57 Unique Lectin Specificities. ACS Chem. Biol..

[74] Maljaars C. E. P., Halkes K. M., de Oude W. L., Haseley S. R., Upton P. J., McDonnell M. B., Kamerling J. P. (2006). Affinity
Determination of Ricinus
communis Agglutinin Ligands Identified from Combinatorial O- and S-,N-Glycopeptide
Libraries. J. Comb. Chem..

[75] Schuster G. B., Platz M. S. (1992). Photochemistry of
phenyl azide. Advances in photochemistry.

[76] Reddy V. S., Rao V. (1992). Modes of binding of
α (1–2) linked manno-oligosaccharides
to concanavalin A. Int. J. Biol. Macromol..

[77] Jeyachandran Y. L., Mielczarski J. A., Mielczarski E., Rai B. (2010). Efficiency of blocking
of non-specific interaction of different proteins by BSA adsorbed
on hydrophobic and hydrophilic surfaces. J.
Colloid Interface Sci..

[78] Al-Husseini J. K., Stanton N. J., Selassie C. R., Johal M. S. (2019). The binding
of drug
molecules to serum albumin: The effect of drug hydrophobicity on binding
strength and protein desolvation. Langmuir.

[79] Smith E. A., Thomas W. D., Kiessling L. L., Corn R. M. (2003). Surface Plasmon
Resonance Imaging Studies of Protein-Carbohydrate Interactions. J. Am. Chem. Soc..

[80] Parera
Pera N., Branderhorst H. M., Kooij R., Maierhofer C., van der Kaaden M., Liskamp R. M. J., Wittmann V., Ruijtenbeek R., Pieters R. J. (2010). Rapid Screening of Lectins for Multivalency Effects
with a Glycodendrimer Microarray. ChemBioChem..

[81] Feldhof M. I., Sperzel S., Bonda L., Boye S., Braunschweig A. B., Gerling-Driessen U. I. M., Hartmann L. (2024). Thiol-selective native
grafting from polymerization for the generation of protein–polymer
conjugates. Chem. Sci..

[82] Olmsted I. R., Kussrow A., Bornhop D. J. (2012). Comparison
of Free-Solution and Surface-Immobilized
Molecular Interactions Using a Single Platform. Anal. Chem..

[83] Ramesh D., Srinivasan M. (1984). Studies on
ring opening of coumarins. Curr. Sci..

[84] Mała P., Siebs E., Meiers J., Rox K., Varrot A., Imberty A., Titz A. (2022). Discovery of N-β-l-Fucosyl
Amides as High-Affinity Ligands for the Pseudomonas aeruginosa Lectin
LecB. J. Med. Chem..

[85] Hayes W., Osborn H. M., Osborne S. D., Rastall R. A., Romagnoli B. (2003). One-pot synthesis
of multivalent arrays of mannose mono-and disaccharides. Tetrahedron.

[86] Wu L., Sampson N. S. (2014). Fucose, Mannose, and β-N-Acetylglucosamine Glycopolymers
Initiate the Mouse Sperm Acrosome Reaction through Convergent Signaling
Pathways. ACS Chem. Biol..

