# Supplementary material for: Azidocoumarin Glycan Probes for Photoinduced Cross-Linking and In Situ Fluorescent Labeling
Source: Bioconjug Chem. 2026 Mar 7;37(3):565–79. doi: 10.1021/acs.bioconjchem.5c00613 (PMC13003445; doi:10.1021/acs.bioconjchem.5c00613)
Supplement: Supplementary file 1 [file bc5c00613_si_001.pdf]

Azidocoumarin-glycan probes for photo-induced crosslinking and *in-situ* fluorescent labeling

Nina Jahnke[1a], Marc D. Driessen[2a], Georgia Partalidou[3], Simon Przetak[1], Ulla I.M. Gerling-Driessen[3], Laura Hartmann[1,3]**

[1] Department of Organic and Macromolecular Chemistry, Heinrich Heine University Duesseldorf, Universitaetsstrasse 1, 40225 Duesseldorf, Germany

[2] University of Cologne, Faculty of Medicine and University Hospital Cologne, Department of Oral, Maxillofacial and Plastic Surgery, Kerpener Str. 62, 50937 Cologne, Germany

[3] Institute for Macromolecular Chemistry, Albert Ludwig University of Freiburg, Stefan-Meier-Strasse 31, 79104 Freiburg, Germany

Email: ulla.gerling-driessen@makro.uni-freiburg.de, laura.hartmann@makro.uni-freiburg.de

[a] These authors contributed equally

Structural characterization

Azidocoumarin (AzC)

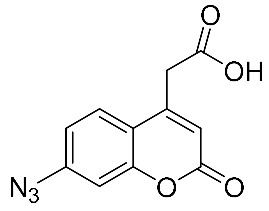

Molecular Weight: 245,19

^{1}H-NMR (300 MHz, DMSO-d_6) δ (ppm) 12.92 (s, 1H), 7.79 (dd, *J* = 8.5, 1.0 Hz, 1H), 7.26 (dd, *J* = 2.2, 1.0 Hz, 1H), 7.21 (dd, *J* = 8.5, 2.3, 1.0 Hz, 1H), 6.51 (d, *J* = 1.1 Hz, 1H), 3.97 (d, *J* = 1.2 Hz, 2H).

^{13}C-NMR (300 MHz, DMSO-*d6*): δ (ppm) 159,50 (C11), 154,00 (C1), 152,90 (C9), 143,26 (C3), 126,96 (C7), 124,16 (C5), 116,73 (C6), 115,53 (C4), 113,16 (C2), 106,78 (C8), 18,06 (C10)

RP-HPLC (5-95 Vol% t H_2O/acetonitrile in 17 min at 25°C, 214 nm): t_r = 8,1 min

ESI-MS calculated $[M+1H]^+$ 246,19, found $[M+1H]^+$ 246,3; $[M+1Na]^+$ 268,1; $[2M+1H]^+$ 513.0

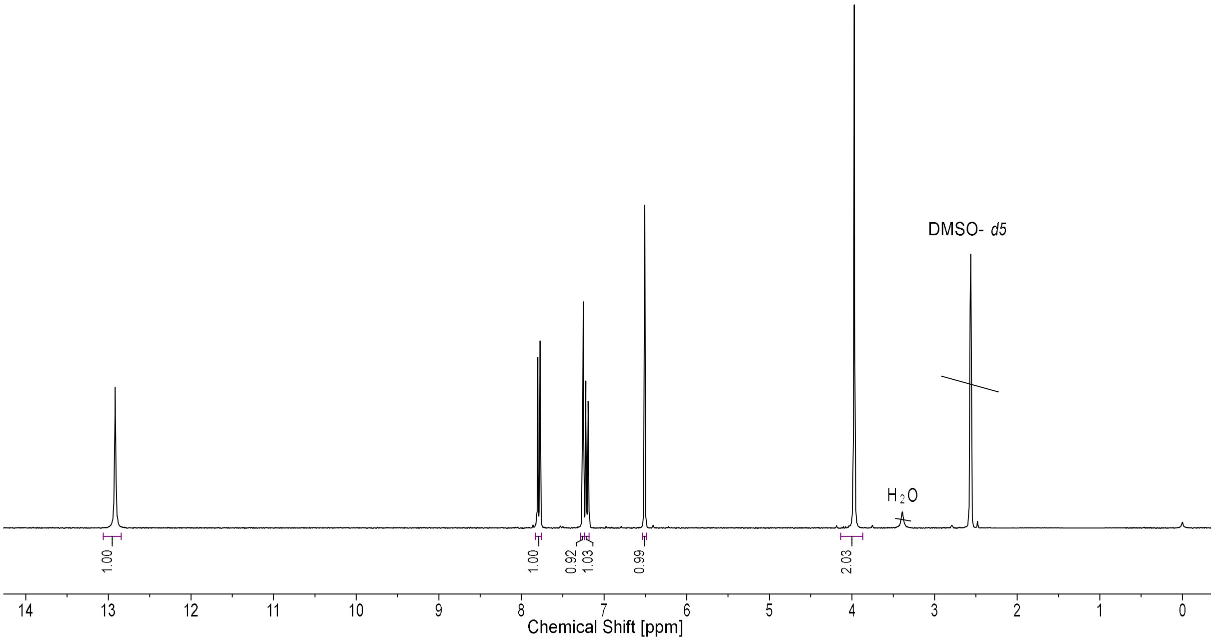

Figure S 1: ^{1}H-NMR (300MHz) spectra of ***AzC.***

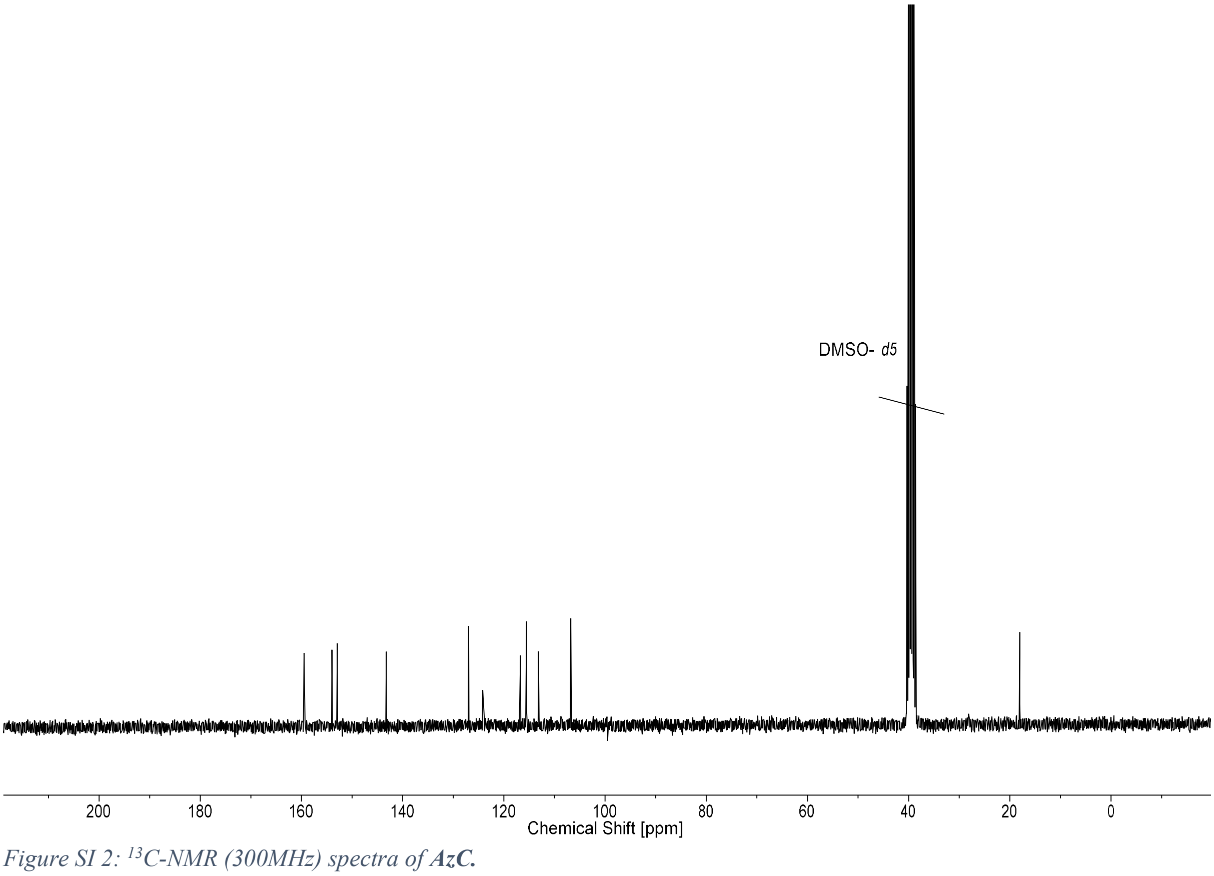

Figure SI 2: ^{13}C-NMR (300MHz) spectra of ***AzC.***

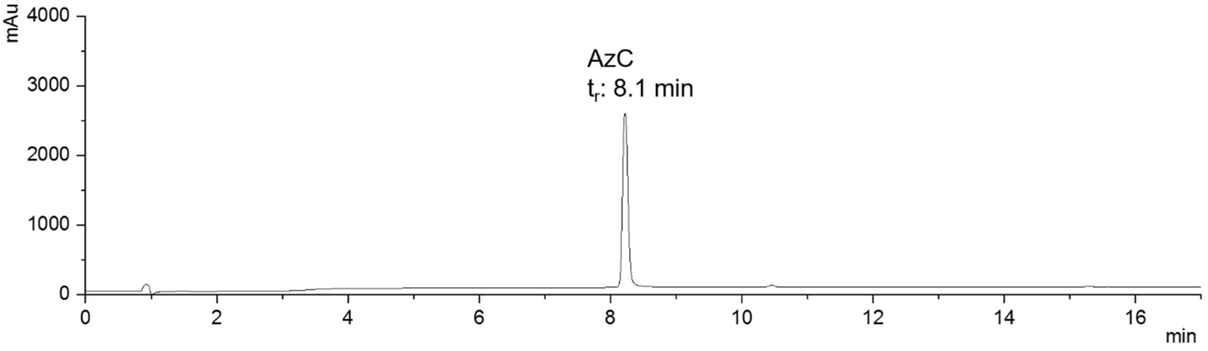

*Figure S 3: **AzC*** detected at t_r = 8,1 min with relative purity >95% by RP-HPLC (*linear gradient from 5-95* Vol% eluent H_2O/acetonitrile in 17 min at 25°C, VWDA1 A Wavelength = 214nm).

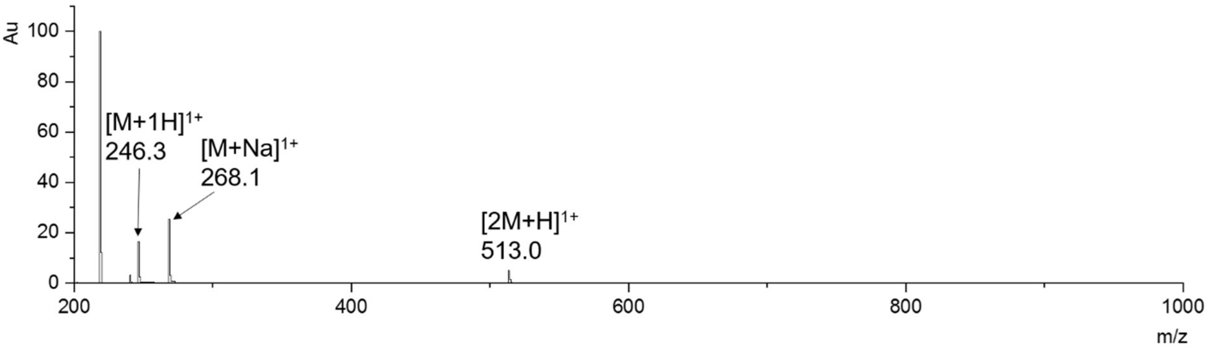

*Figure S 4: **AzC*** analyzed with ESI-MS in a m/z range of 200-2000.

Azidocoumarin-Mannose (AzCMan)

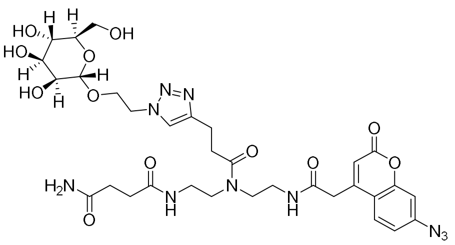

Molecular Weight: 758,75

^{1}H-NMR (300 MHz, Deuterium Oxide) δ (ppm): δ 7.71 (d, *J* = 28.9 Hz, 1H), 7.57 (dd, *J* = 24.9, 9.1 Hz, 1H), 7.10 – 6.85 (m, 2H), 6.31 (d, *J* = 13.0 Hz, 1H), 4.49 (dd, *J* = 10.5, 5.1 Hz, 1H), 3.97 (qt, *J* = 13.7, 7.0 Hz, 2H), 3.89 – 3.09 (m, 20H), 3.07 – 2.60 (m, 5H), 2.59 – 2.22 (m, 8H).

RP-HPLC (5-95 Vol% t H_2O/acetonitrile in 17 min at 25°C, 214 nm): t_r = 5.8 min

ESI-MS calculated $[M+1H]^+$ 759.75, found $[M+2H]2^+$ 380.2; $[M+H]^+$ 759.2

HR-ESI-MS: for $C_{32}H_{43}N_{20}O_{12}$ m/z $[M+1H]^{1+}$ calcd.: 759.30, found: 759.3078, mass accuracy -0.2 ppm

IR: υ($-N_3$) 2160-2120cm^{-1}; found 2121 cm^{-1}

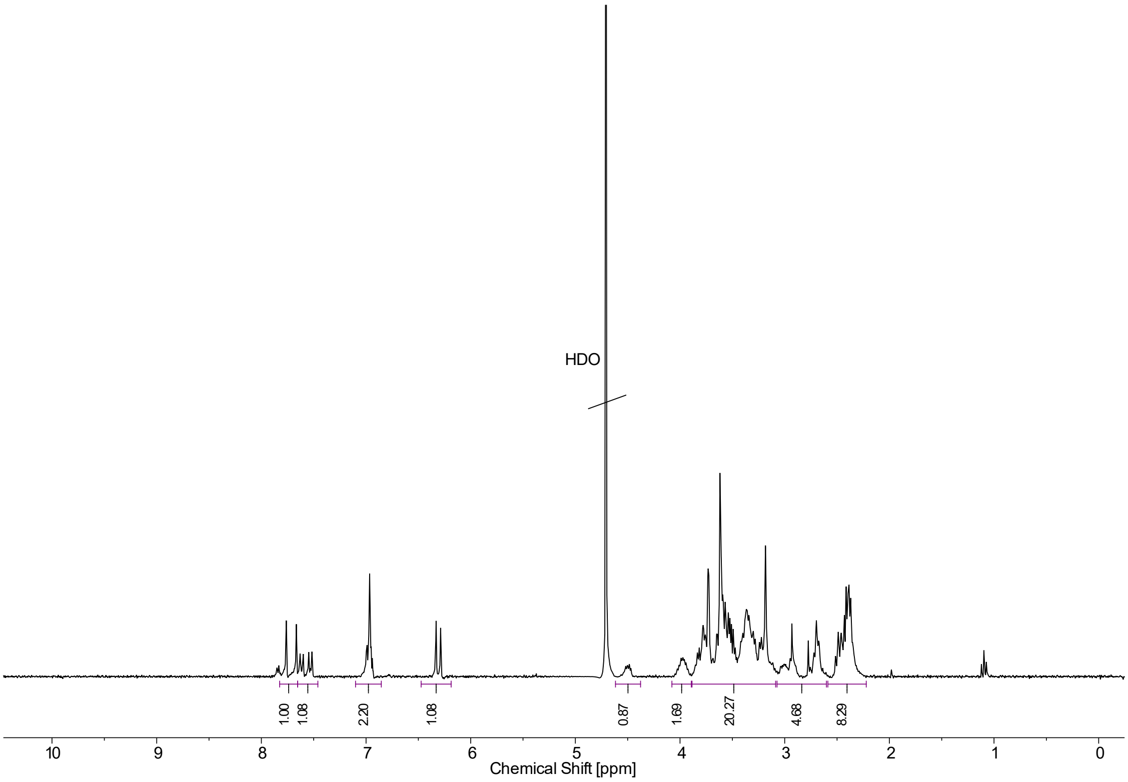

*Figure S 5: ^{1}H-NMR (300MHz) spectra of **AzCMan.***

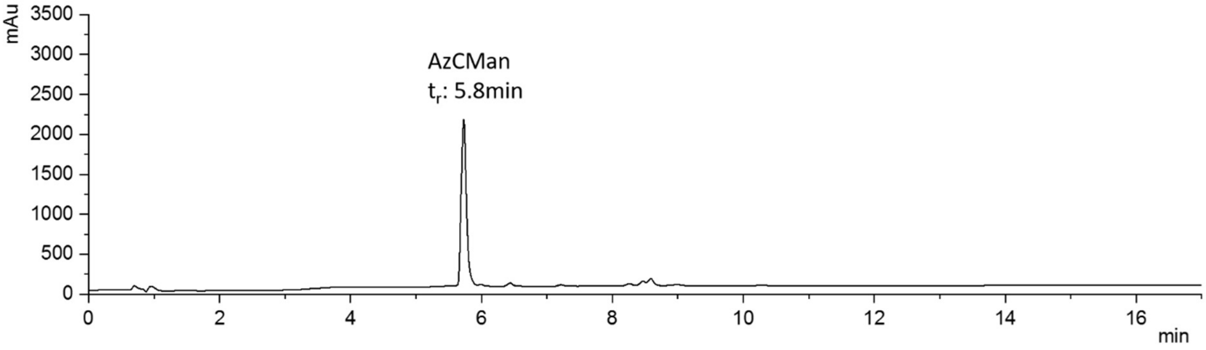

*Figure S 6:**AzCMan*** detected at t_r = 5.8 min with relative purity >95% by RP-HPLC (*linear gradient from 5-95* Vol% eluent H_2O/acetonitrile in 17 min at 25°C, VWDA1 A Wavelength = 214nm).

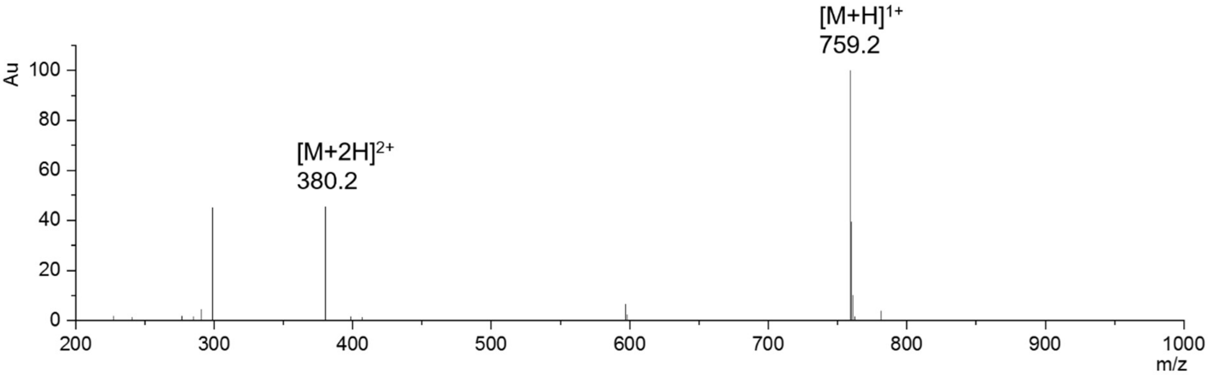

*Figure S 7:**AzCMan*** analyzed with ESI-MS in a m/z range of 200-2000.

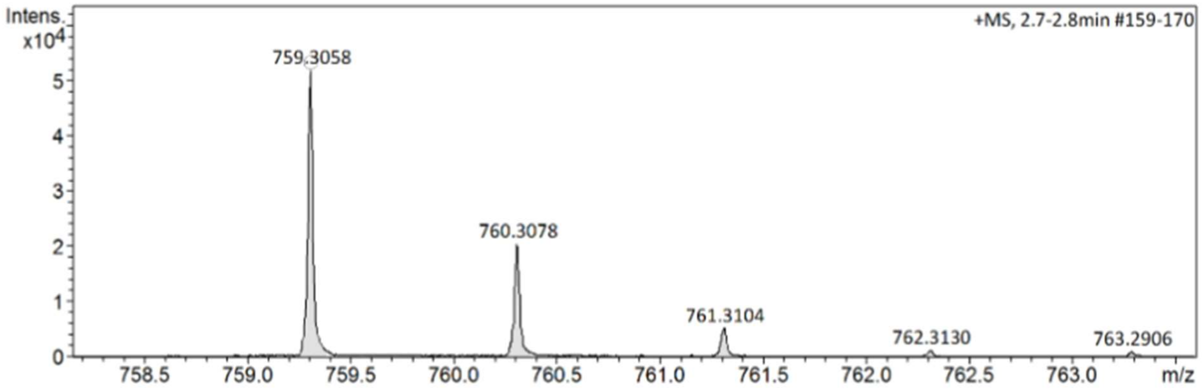

*Figure S 8:**AzCMan** analyzed with HR-ESI.*

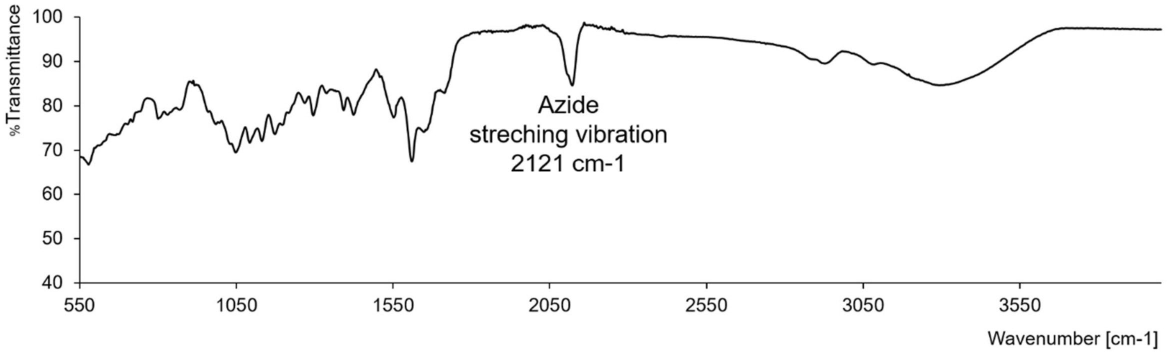

*Figure S 9: IR spectrum of **AzCMan.***

Azidocoumarin-Galactose (AzCGal)

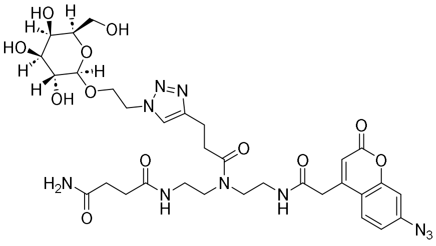

^{1}H-NMR (300 MHz, Deuterium Oxide) δ (ppm): δ 7.75 (d, J = 26.5 Hz, 1H), 7.57 (dd, J = 27.4, 8.7 Hz, 1H), 7.10 – 6.93 (m, 2H), 6.31 (d, J = 13.9 Hz, 1H), 4.51 (dd, J = 9.8, 4.9 Hz, 1H), 4.37 – 4.06 (m, 2H), 3.98 (dt, J = 12.0, 6.2 Hz, 1H), 3.89 – 3.05 (m, 24H), 2.84 – 2.61 (m, 3H), 2.59 – 2.26 (m, 7H).

RP-HPLC (5-95 Vol% t H_2O/acetonitrile in 17 min at 25°C, 214 nm): t_r = 5.8 min

ESI-MS calculated $[M+1H]^+$ 759.75, found $[M+2H]^{2+}$ 380.2; $[M+H]^+$ 759.2

HR-ESI-MS: for $C_{32}H_{43}N_{20}O_{12}$ m/z $[M+1H]^{1+}$ calcd.: 759.30, found: 759.3049, mass accuracy 1.0 ppm

IR: υ($-N_3$) 2160-2120cm^{-1}; found 2120 cm^{-1}

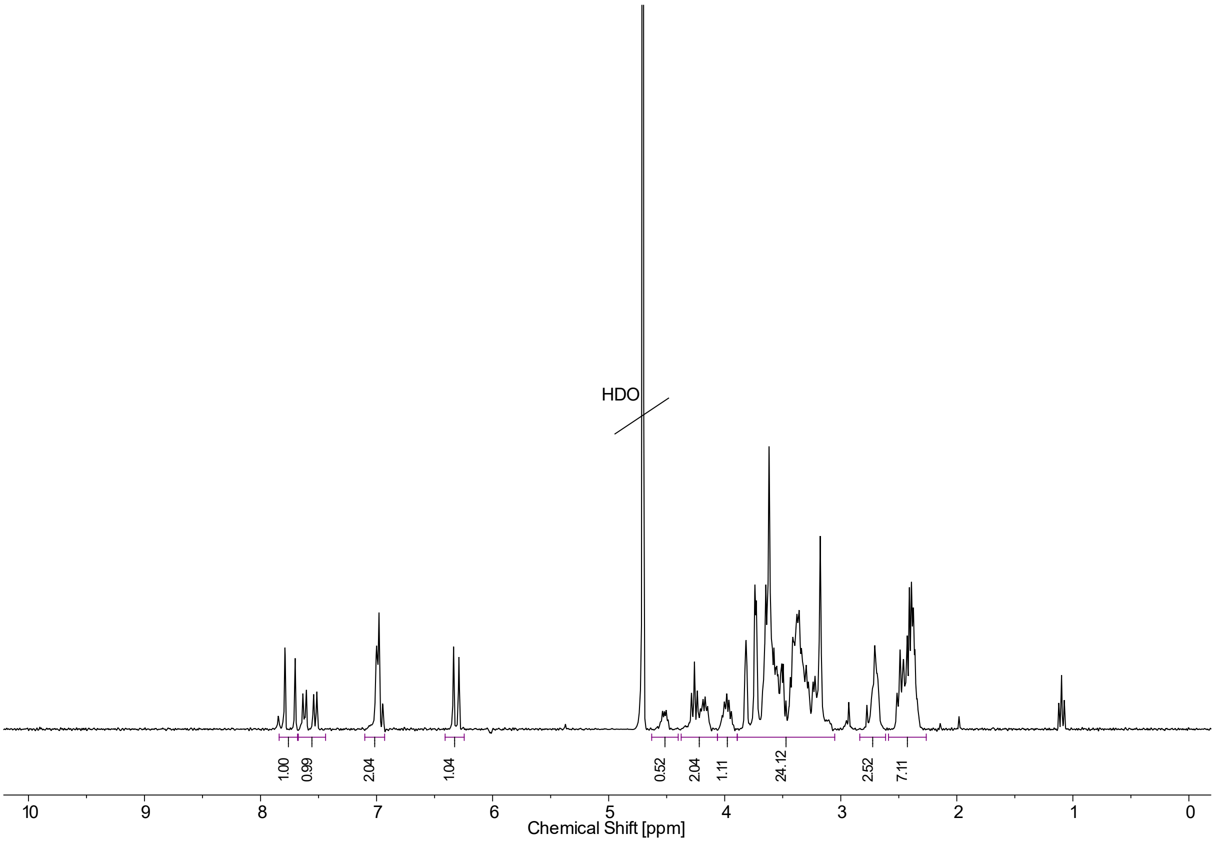

*Figure S 10: ^{1}H-NMR (300MHz) spectra of **AzCGal.***

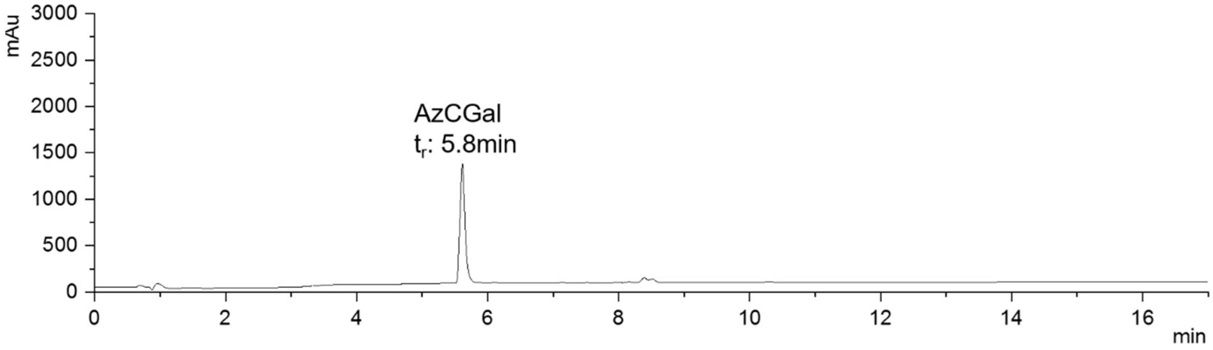

*Figure S 11:**AzCGal*** detected at t_r = 5.8 min with relative purity >95% by RP-HPLC (*linear gradient from 5-95* Vol% eluent H_2O/acetonitrile in 17 min at 25°C, VWDA1 A Wavelength = 214nm).

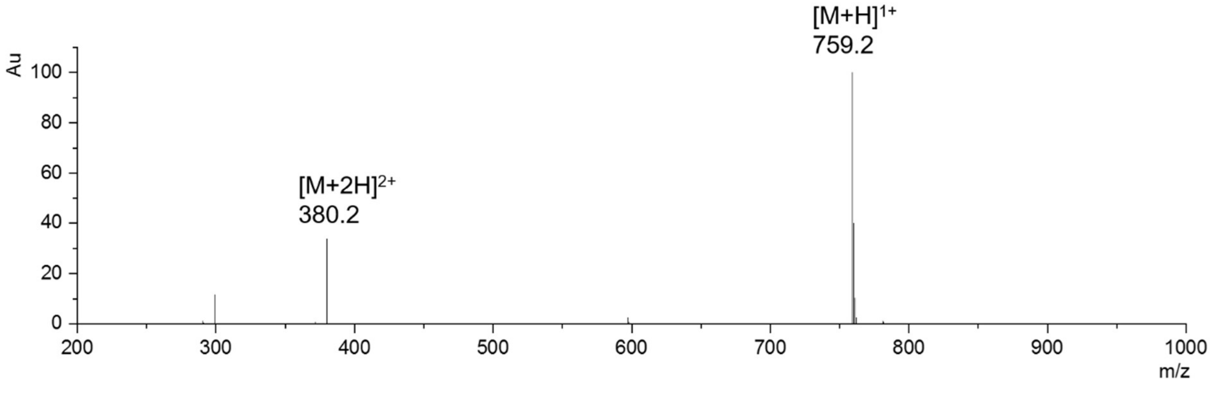

*Figure S 12:**AzCGal*** analyzed with ESI-MS in a m/z range of 200-2000.

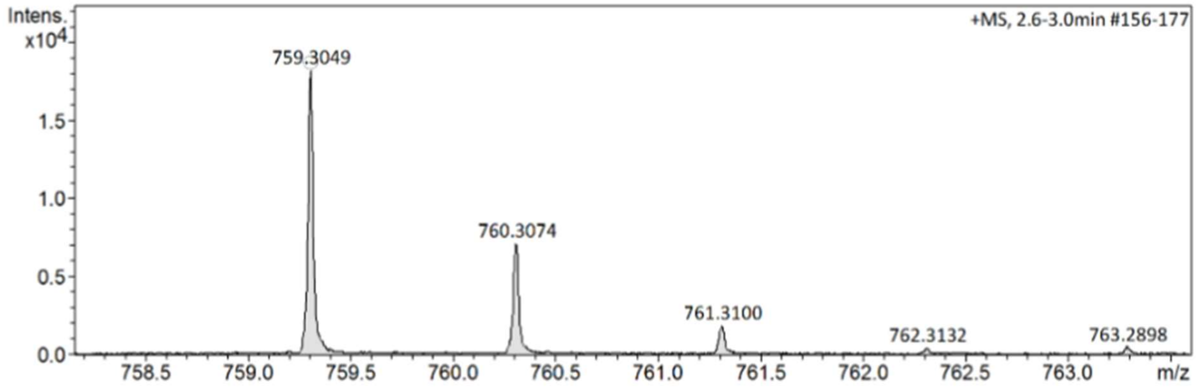

*Figure S 13:**AzCGal** analyzed with HR-ESI.*

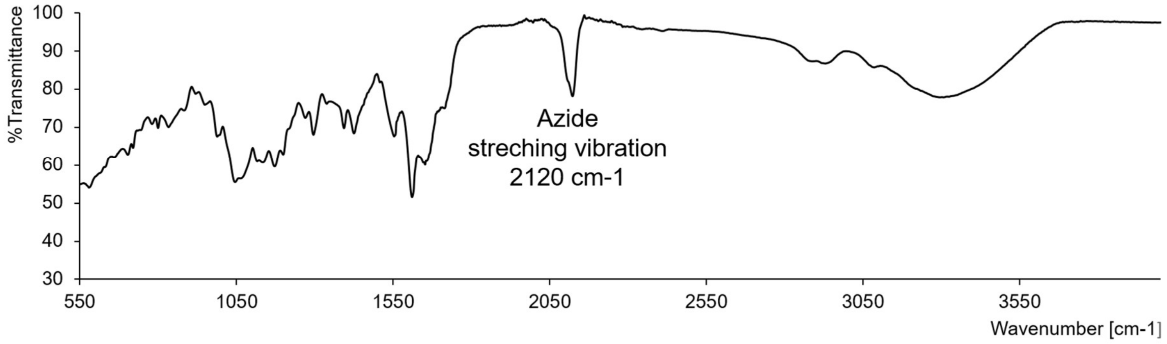

*Figure S 14: IR spectrum of **AzCGal.***

Biotin-Lys-TDS-Mannose-Azidocoumarin (AzManB)

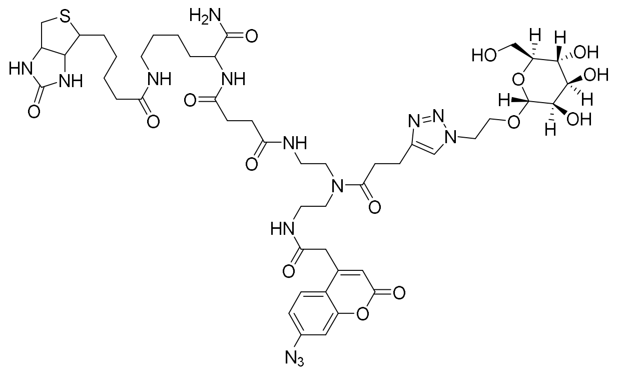

Molecular Weight: 1112.47

^{1}H-NMR (400 MHz, Deuterium Oxide) δ (ppm): δ 7.84 – 7.60 (m, 3H), 7.57 (m, 1H), 6.31 (d, *J* = 13.9 Hz, 1H), 4.61 – 4.56 (m, 2H), 4.42 – 4.36 (m, Hz, 1H), 3.92 – 3.83 (m, 16H), 3.33 – 3.05 (m, 8H), 2.82 – 2.72 (m, 3H), 2.67 – 2.35 (m, 8H), 2.27 – 2.19 (m, 2H), 1.93-1.18 (m, 16H).

^{13}C-NMR (400 MHz, Deuterium Oxide) δ (ppm): 177,19 (C17), 176,54 (C17), 174,82 (C16), 174,77 (C10), 174,72 (C34), 174,66 (C23), 165,26 (C1), 163,10 (C27), 150,80 (C25), 146,49 (C28), 144,73 (C30), 126,61 (C37), 123,79 (C32), 116,28 (C31), 115,84 (C38), 114,90 (C29), 114,59 (C33), 107,07 (C26), 99,46 (C41), 72,77 (C42), 70,44 (C44), 69,89 (C45), 66,37 (C43), 65,40 (C3), 65,37 (C40), 62,06 (C15), 60,67 (C2), 60,63 (C46), 60,21 (C5), 55,37 (C21), 53,53 (C39), 49,91 (C24), 39,66 (C4), 38,90 (C11) 35,45 (C22), 31,88 (C20), 31,66 (C9), 30,54 (C35), 30,41 (C19), 27,86 (C18), 27,82 (C14), 27,64 (C12), 27,64 (C7), 25,17 (C36), 22,52 (C8), 20,58(C6), 20,40 (C13)

RP-HPLC (5-50 Vol% t H_2O/acetonitrile in 20 min at 25°C, 214 nm): t_r = 10.91 min

ESI-MS calculated $[M+1H]^+$ 1113,6; found $[M+1H]^+$ 1113,6; calculated $[M+2H]^+$ 557,6; found $[M+1H]^+$ 557,6

HR-ESI-MS: for $C_{48}H_{68}N_{14}O_{15}S$ sm/z $[M+1H]^{1+}$ calcd.: 1113,47, found: 1113,4807, mass accuracy 1.0 ppm; for $NaC_{48}H_{67}N_{14}O_{15}S$ sm/z $[M+1Na]^{1+}$ calcd.: 1135,47, found: 1135,4629, mass accuracy 1.0 ppm

IR: υ($-N_3$) 2160-2120cm^{-1}; found: 2117 cm^{-1}

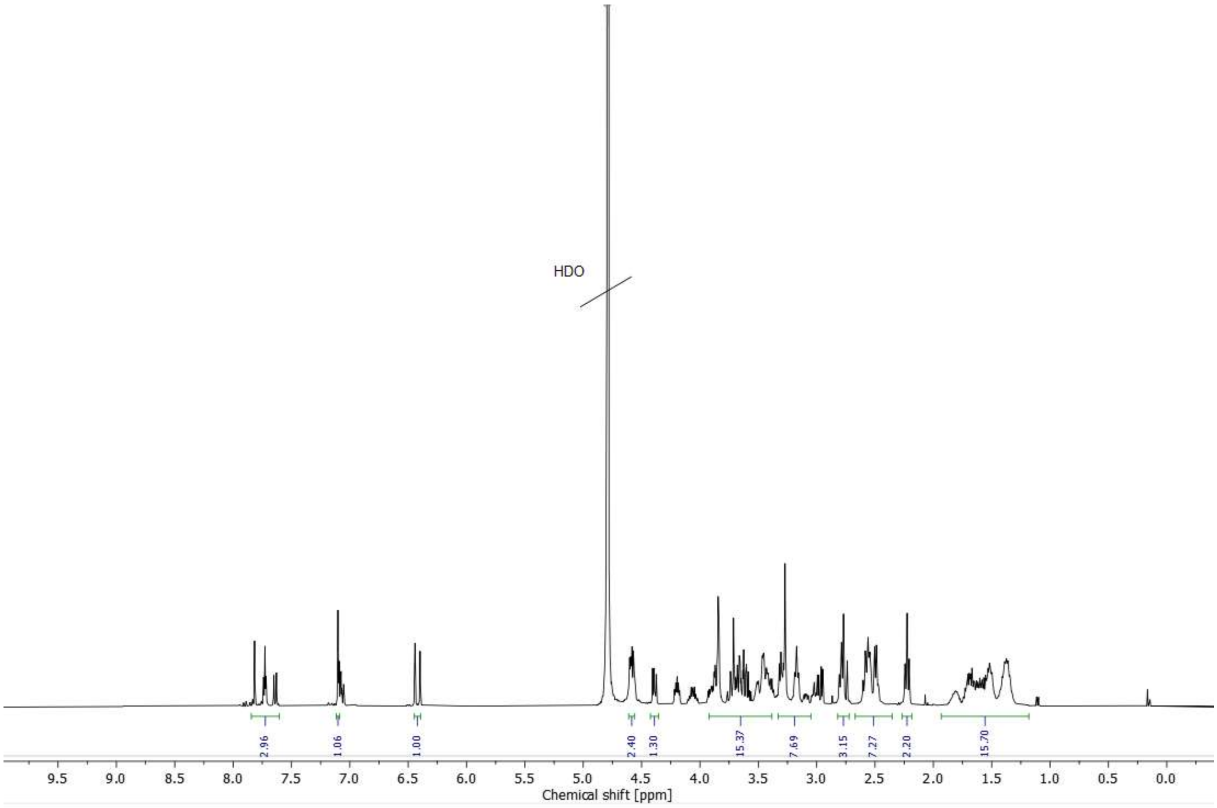

Figure S 15: ^{1}H-NMR (400MHz) spectra of ***AzManB****.*

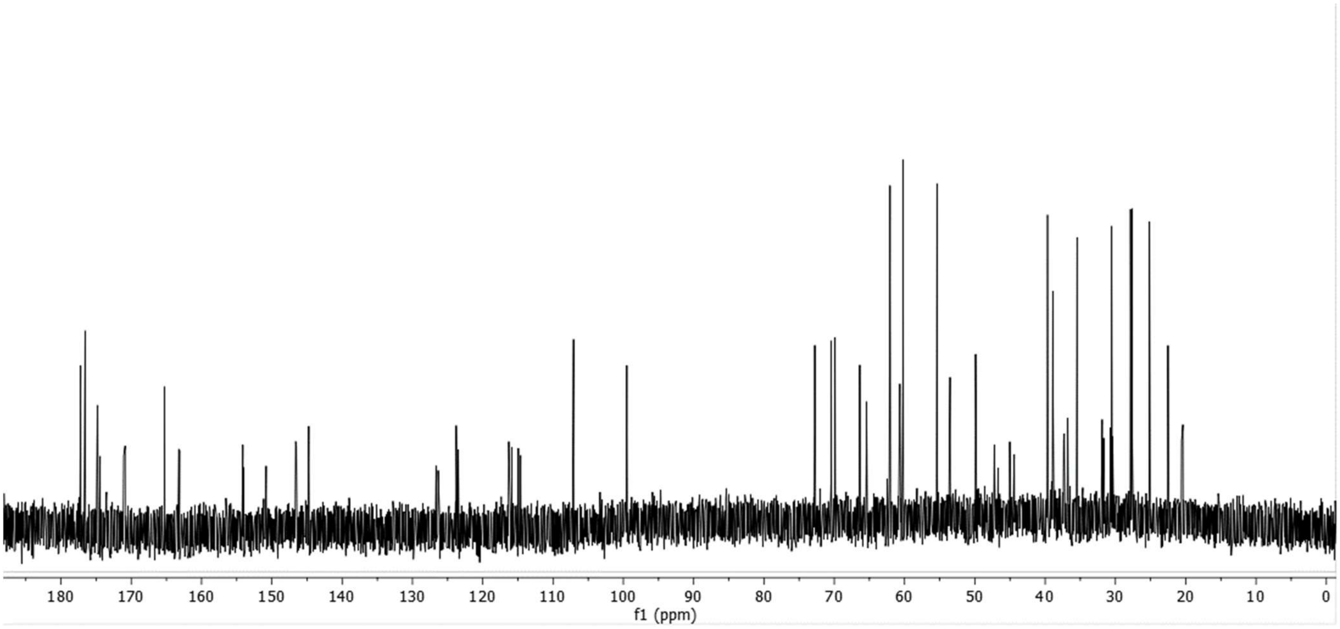

Figure S 16: ^{13}C-NMR (400MHz) spectra of ***AzManB.***

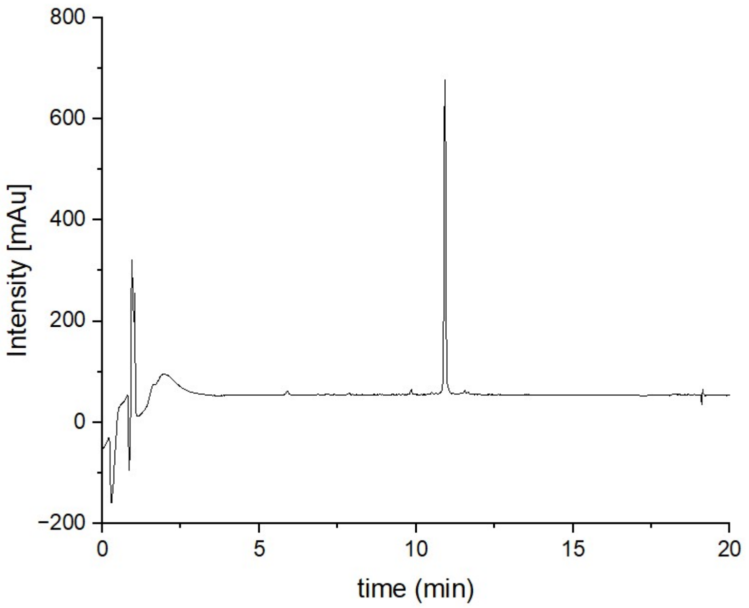

*Figure S 17:**AzManB*** detected at t_r = 10,91 min with relative purity >95% by RP-HPLC (*linear gradient from 1-50* Vol% eluent H_2O/acetonitrile in 20 min at 25°C, VWDA1 A Wavelength = 214nm).

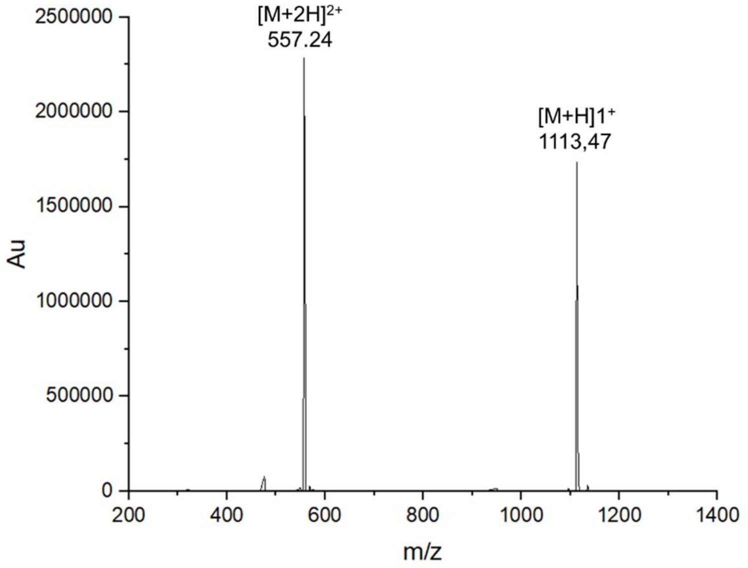

*Figure S 18: **AzManB*** analyzed with ESI-MS in a m/z range of 200-2000.

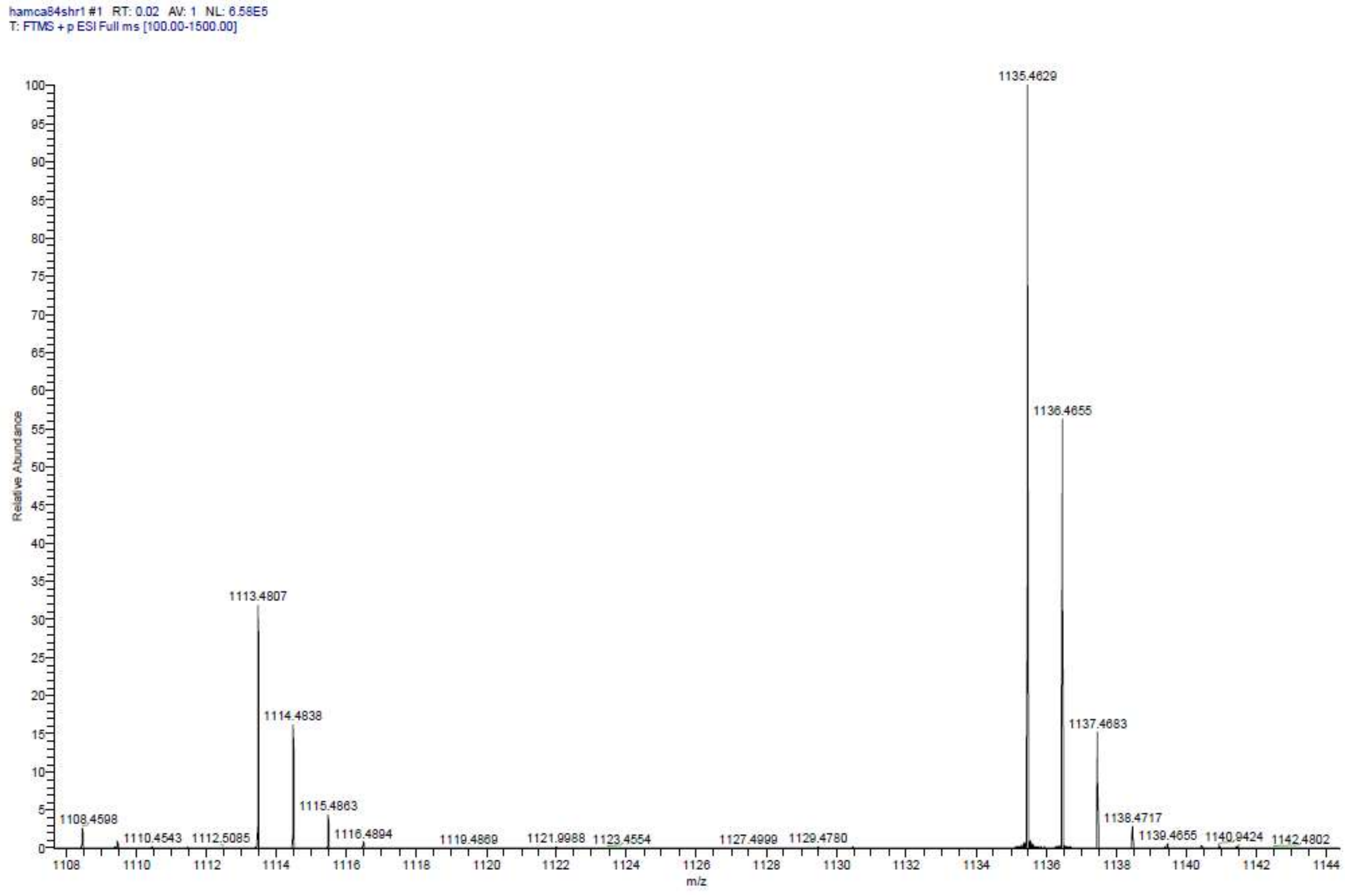

*Figure S 19: **AzManB** analyzed with HR-ESI.*

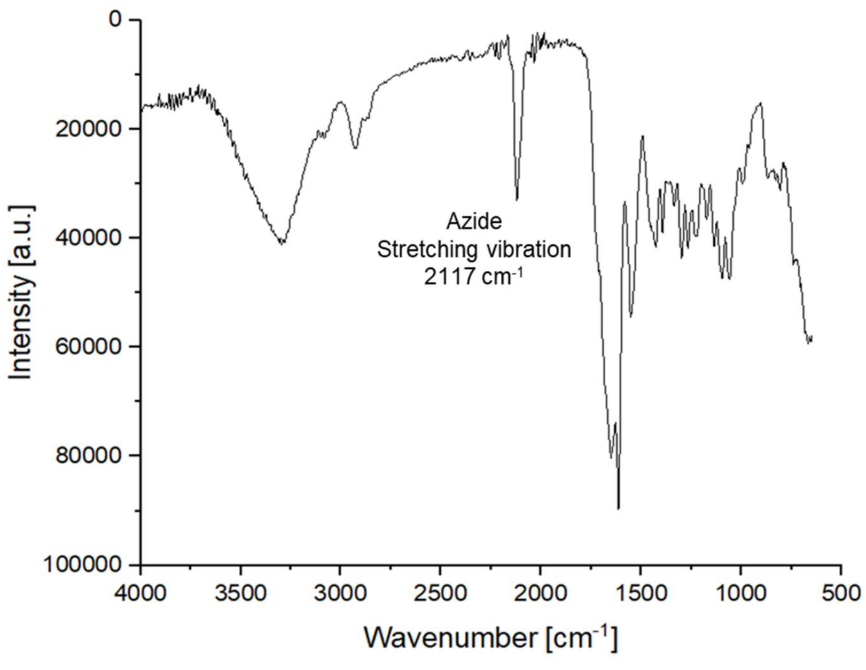

*Figure S 20: IR spectrum of **AzManB.***

Absorption and fluorescence spectra of AzC

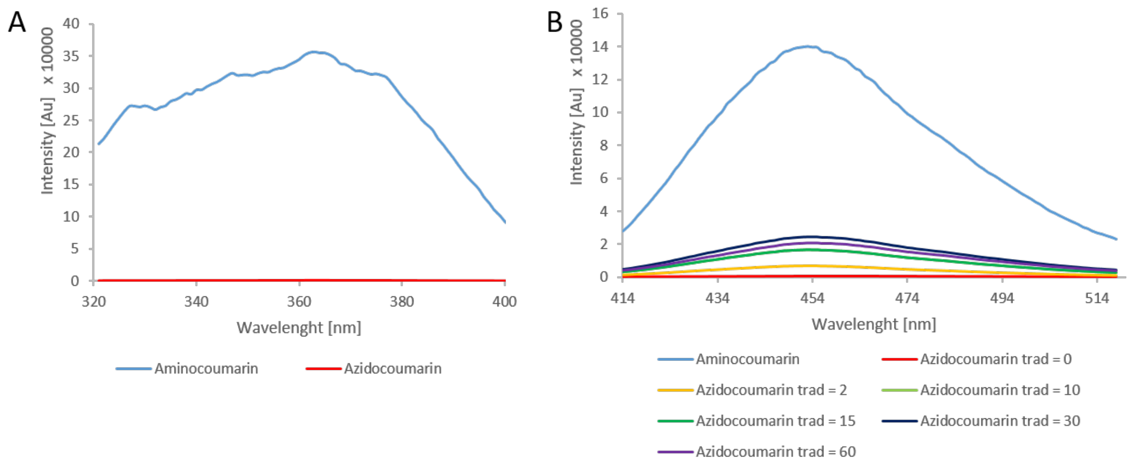

Figure S 21: A) Extinction of azidocoumarin (red) and aminocoumarin (blue) in comparison. B) Fluorescence measurements of azidocoumarin after different durations of irradiation (trad=0 to trad = 60 min) at 365 nm in comparison to aminocoumarin.

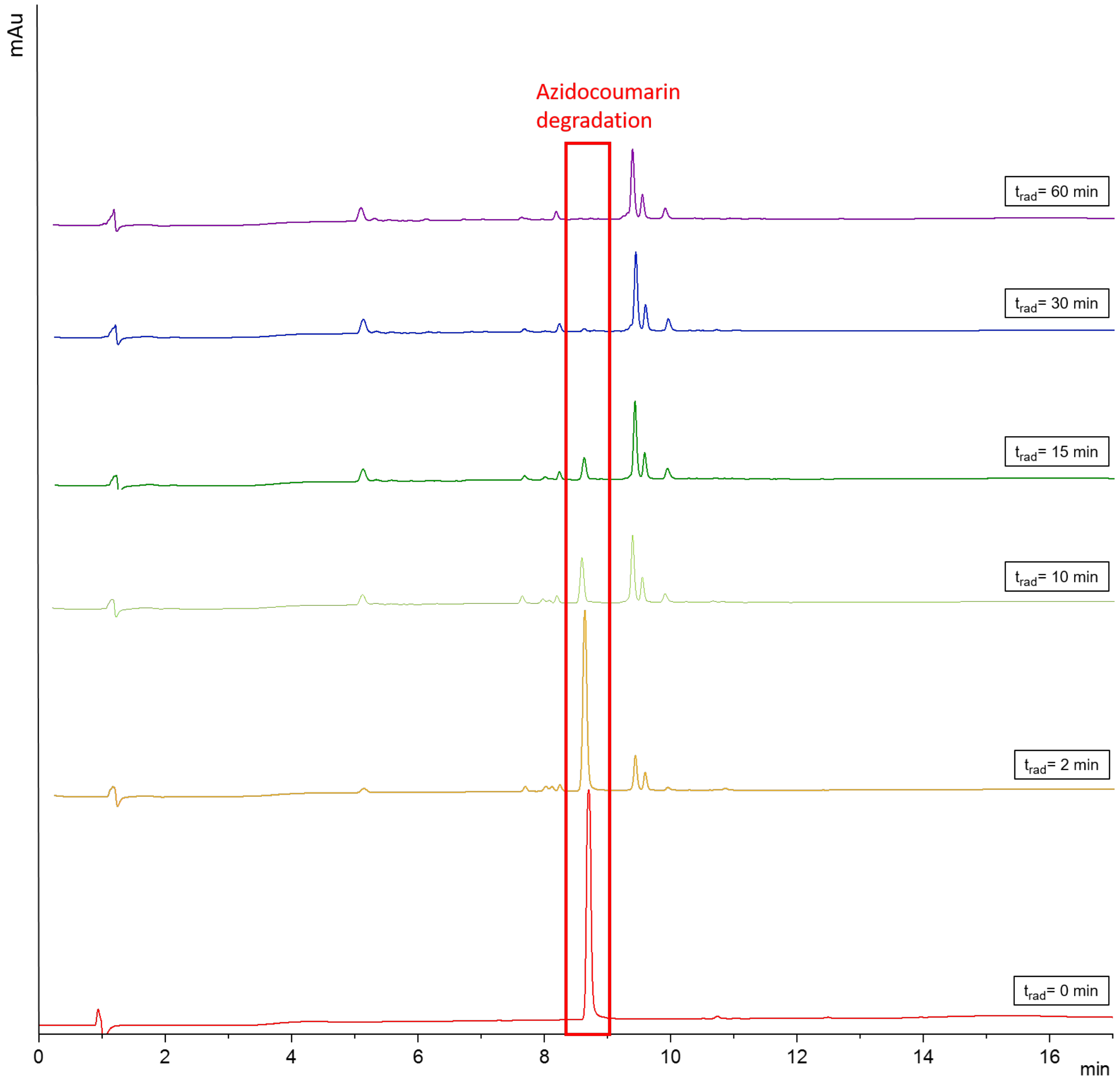

Figure S 22: RP-HPLC of AzC after different irradiation times showing reduce of the AzC signal due to degradation.

MALDI-TOF-MS experiments

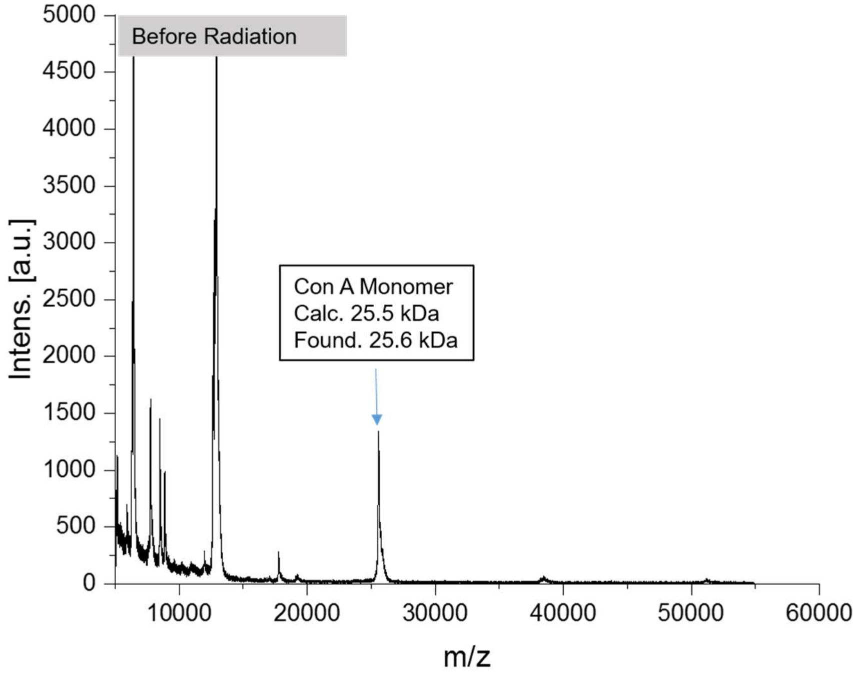

Figure S 23: MALDI-TOF-MS of *ConA and **AzCMan** before irradiation* in a m/z range of 20000-60000 using HCCA as matrix in a compound to matrix ratio of 1:2.

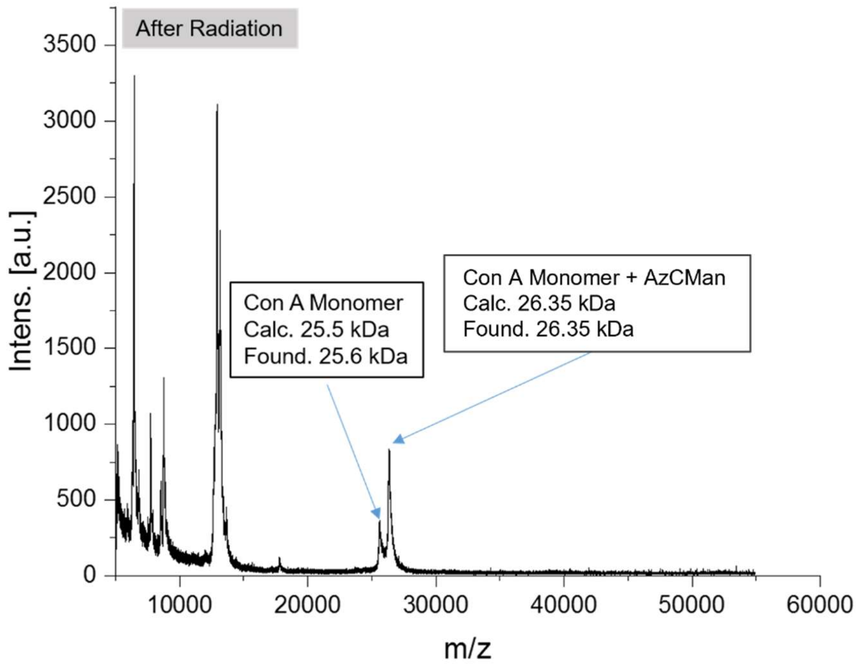

Figure S 24: MALDI-TOF-MS of *ConA and **AzCMan** after radiation* in a m/z range of 20000-60000 using HCCA as matrix in a compound to matrix ratio of 1:2.

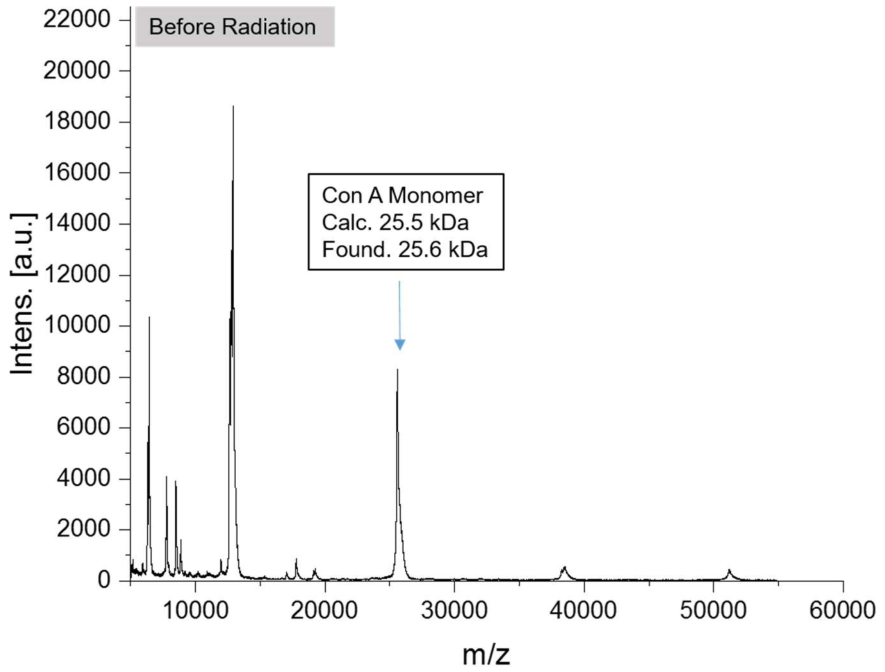

Figure S 25: MALDI-TOF-MS of *ConA and* ***AzCGal*** *before radiation* in a m/z range of 20000-60000 using HCCA as matrix in a compound to matrix ratio of 1:2.

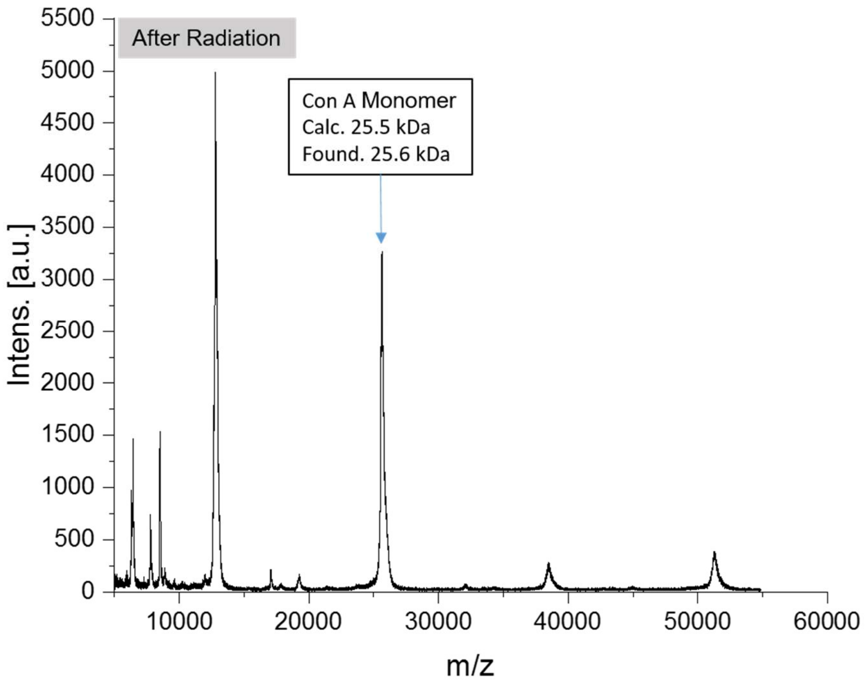

Figure S 26: MALDI-TOF-MS of ConA and ***AzCGal*** *after radiation in a m/z range of 20000-60000 using HCCA as matrix in a compound to matrix ratio of 1:2.*

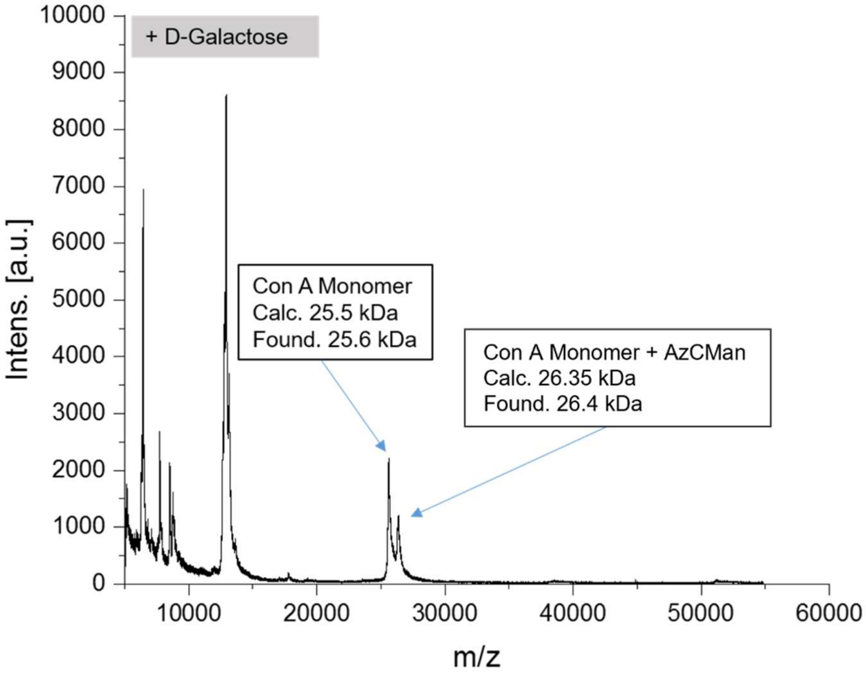

Figure S 27: MALDI-TOF-MS of *ConA and* ***AzCMan*** *+ D-Galactose after radiation* in a m/z range of 20000-60000 using HCCA as matrix in a compound to matrix ratio of 1:2.

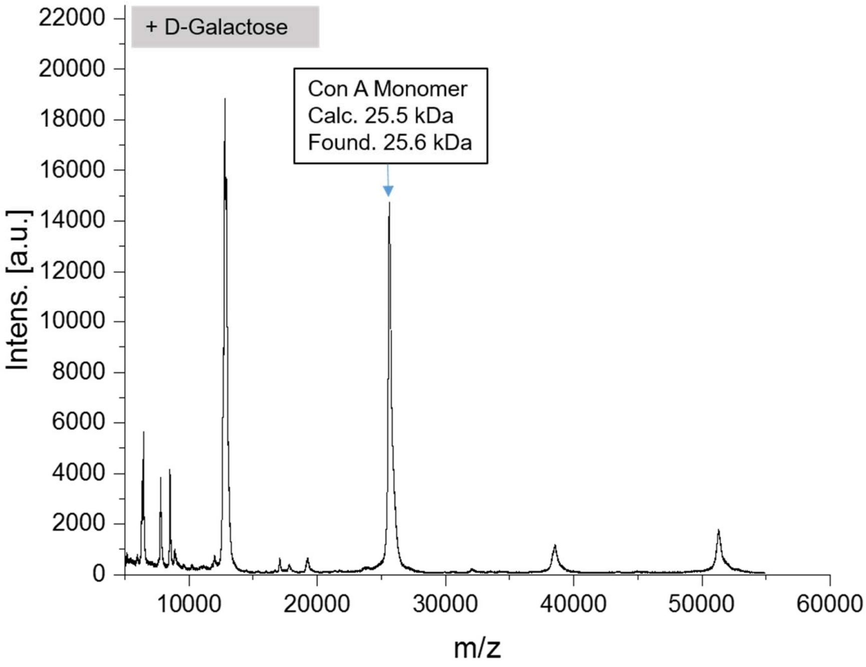

Figure S 28. MALDI-TOF-MS of *ConA and* ***AzCGal*** *+ D-Galactose after radiation* in a m/z range of 20000-60000 using HCCA as matrix in a compound to matrix ratio of 1:2.

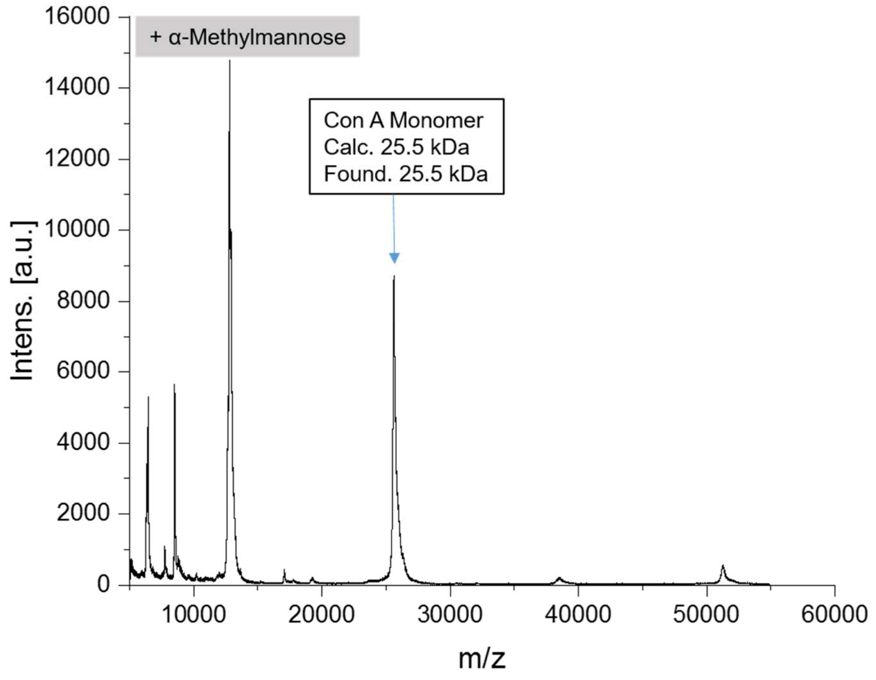

*Figure S 29: MALDI-TOF-MS of ConA and **AzCMan** + Methylmannose after radiation in a m/z range of 20000-60000 using HCCA as matrix in a compound to matrix ratio of 1:2.*

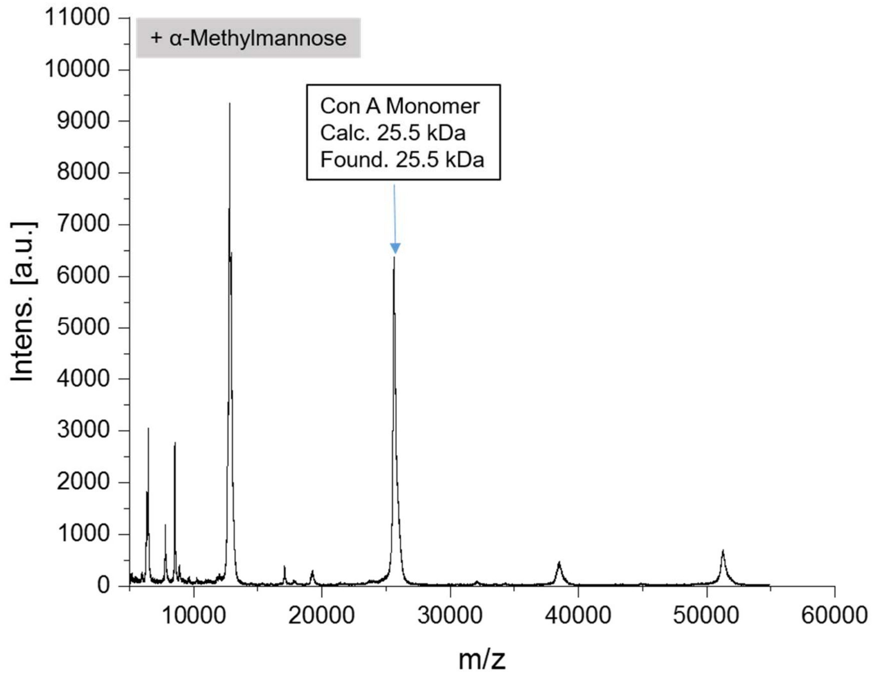

*Figure S 30: MALDI-TOF-MS of ConA and **AzCGal** + Methylmannose after radiation in a m/z range of 20000-60000 using HCCA as matrix in a compound to matrix ratio of 1:2.*

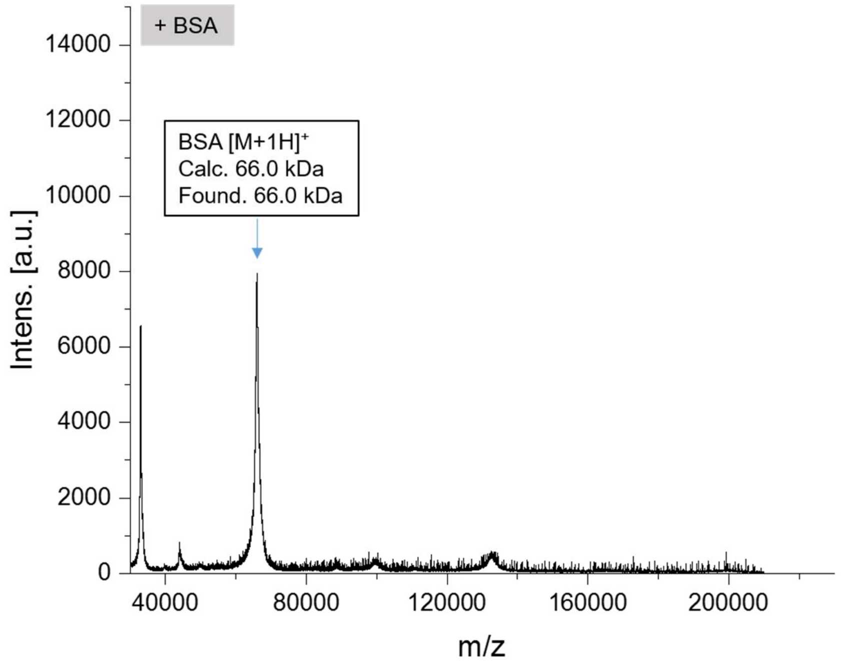

Figure S 31: MALDI-TOF-MS of *BSA* + ***AzCMan*** *after radiation* in a m/z range of 50000-200000 using HCCA as matrix in a compound to matrix ratio of 1:2.

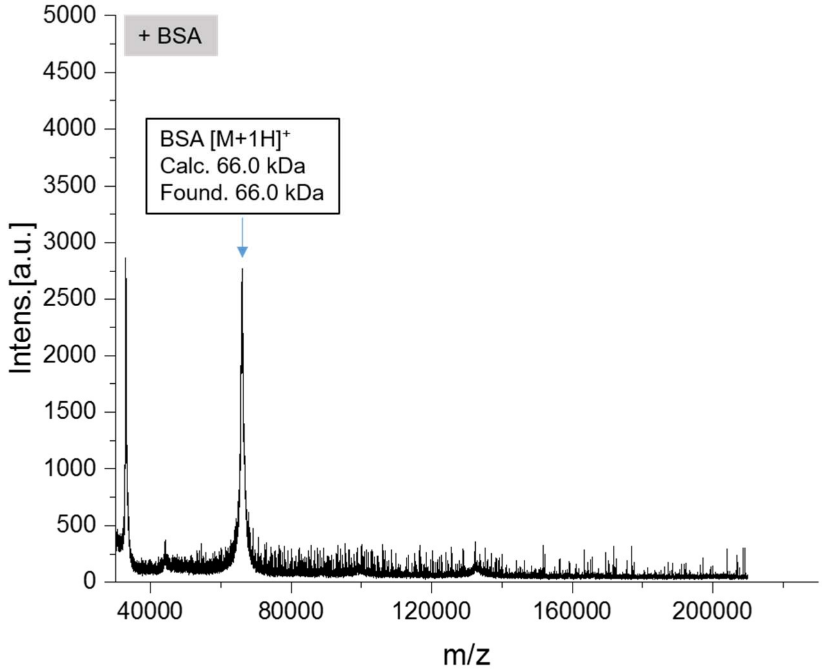

Figure S 32: MALDI-TOF-MS of *BSA and* ***AzCGal*** *after radiation* in a m/z range of 50000-200000 using HCCA as matrix in a compound to matrix ratio of 1:2.

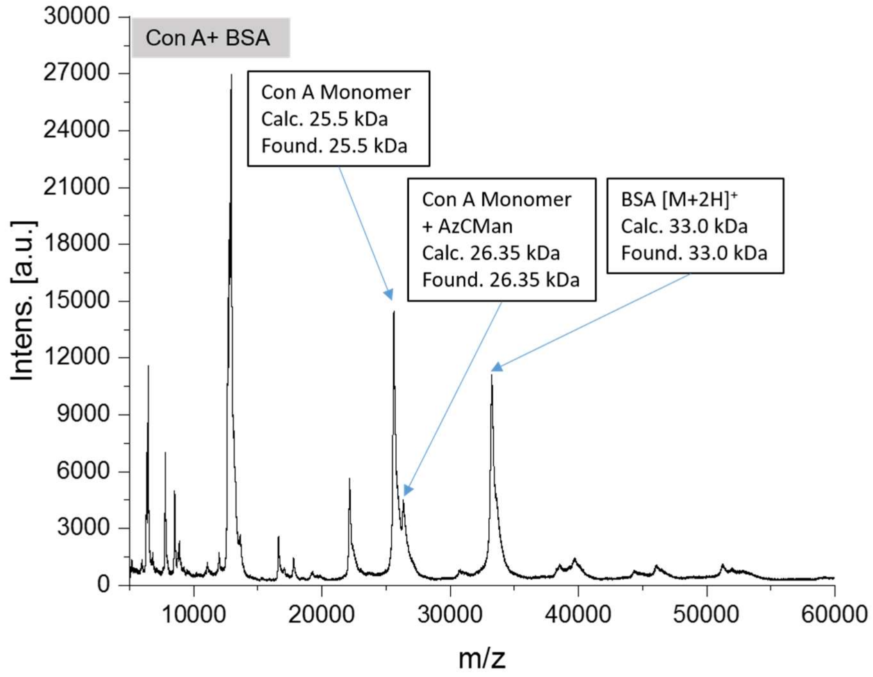

*Figure S 33: MALDI-TOF-MS of ConA and **AzCMan** + BSA after radiation in a m/z range of 20000-60000 using HCCA as matrix in a compound to matrix ratio of 1:2.*

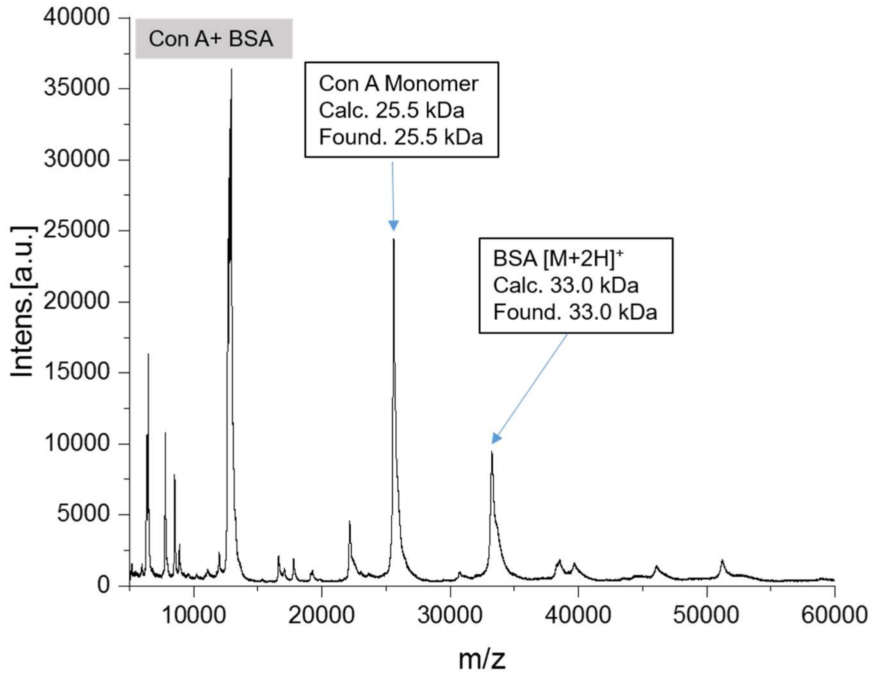

*Figure S 34: MALDI-TOF-MS of ConA and **AzCGal** + BSA after radiation in a m/z range of 20000-60000 using HCCA as matrix in a compound to matrix ratio of 1:2.*

Fluorescence spectra

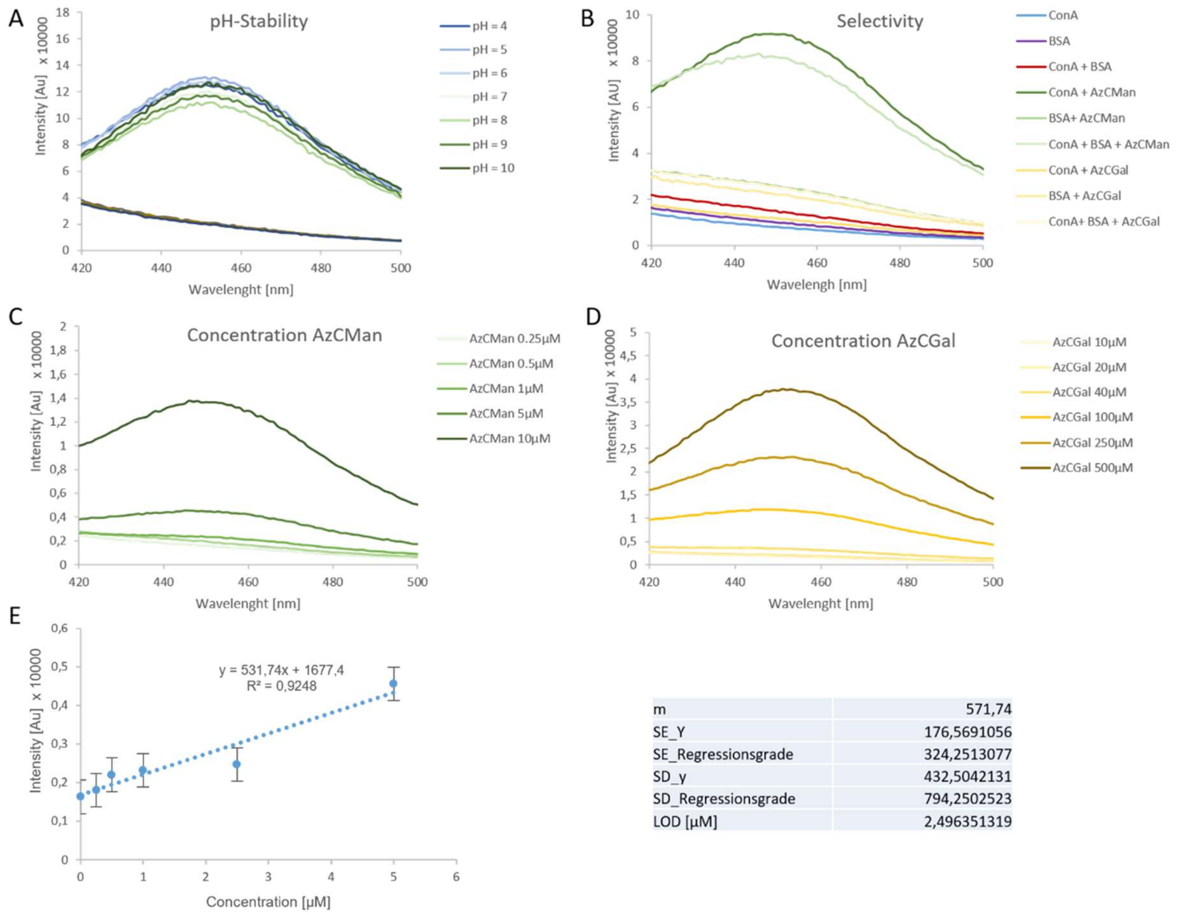

m	571,74
SE_Y	176,5691056
SE_Regressionsgrade	324,2513077
SD_y	432,5042131
SD_Regressionsgrade	794,2502523
LOD [µM]	2,496351319

Figure S 35:Microplate Reader fluorescence spectra. A) pH stability of AzCMan-ConA conjugate. B) Selective binding and fluorogenic properties of AzC PAL probes in presence of different lectins and proteins. C) Fluorogenic properties of AzCMan at different concentrations. D) Fluorogenic properties of AzCGal at different concentrations. E) Calculated limit of detection for AzCMan.

Additional SDS-PAGE images

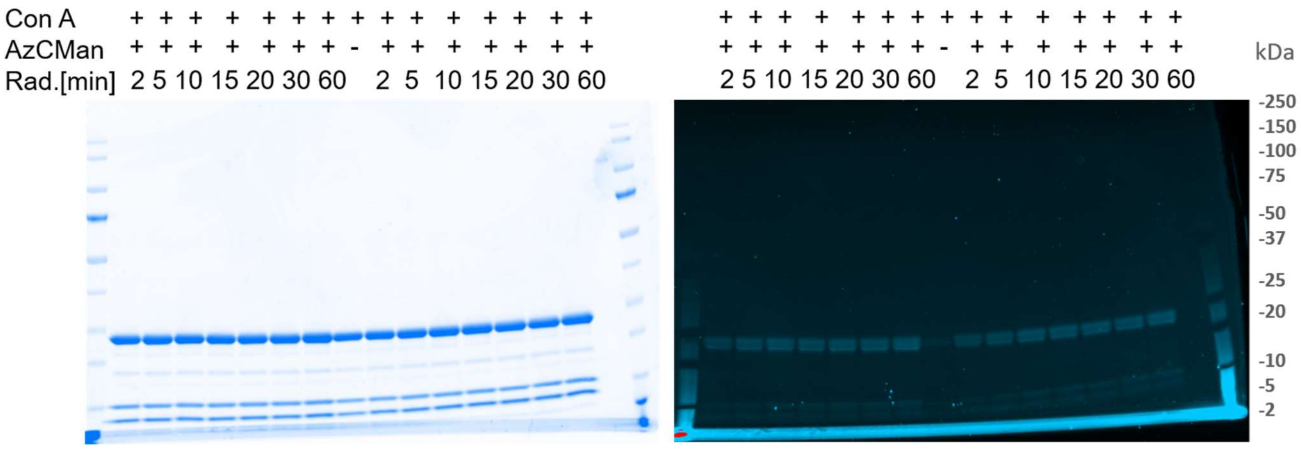

Figure S 36: SDS-PAGE showing relative ConA crosslinking of AzCMan after different durations of irradiation.

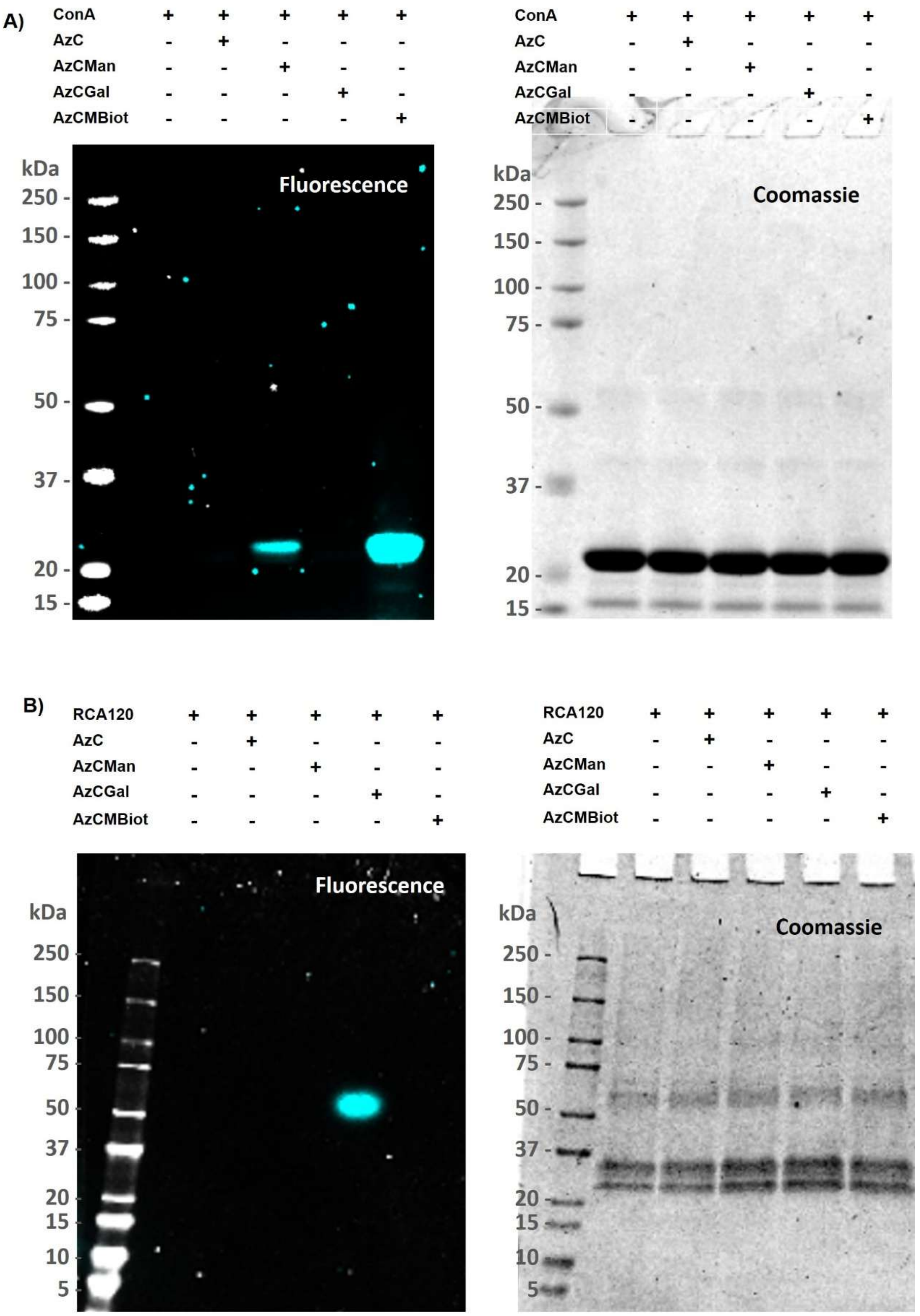

Figure S 37: A) SDS-PAGE of ConA incubated with AzC probes after 15 min irradiation at 365 nm; Left image shows AzC fluorescence; right image shows Coomassie stain of the same gel. B) SDS-PAGE of RCA_{120} incubated with AzC probes after 15 min irradiation at 365 nm. Left image shows AzC fluorescence, right image shows Coomassie stain of the same gel.

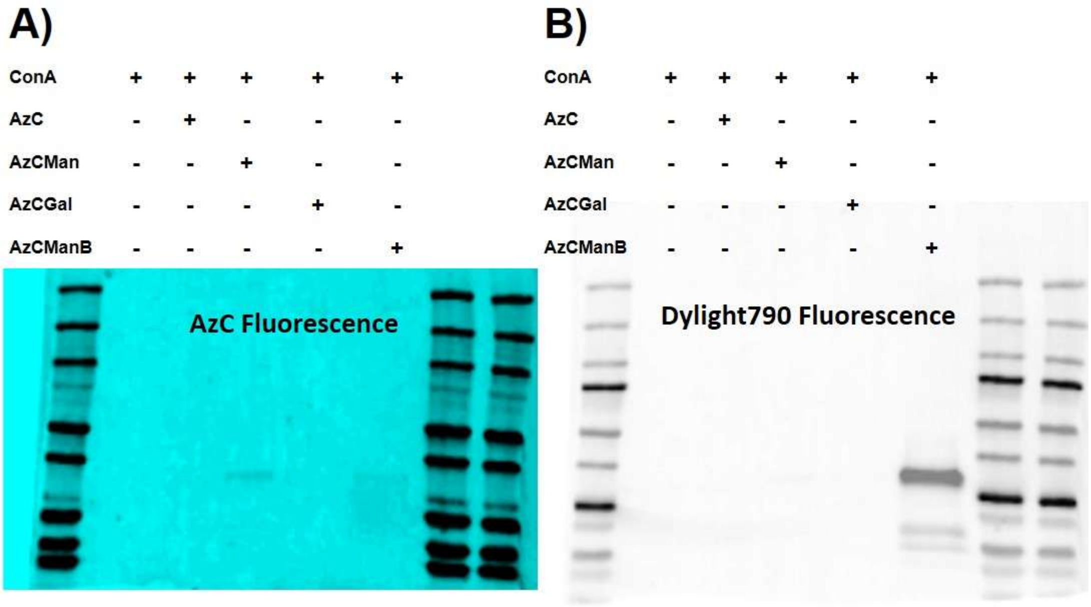

Figure S 38: A) Western blot of ConA incubated with AzC building block and AzC glycan PAL probes 15 min irradiation at 365 nm; A) WB showing coumarin fluorescence (fluorescence was measured in the Cy5 channel). B) Image of the same blot after incubation with streptavidin-dylight 790

Additional fluorescence microscopy images

Triplicate measurements fixed cells incubated with AzC probes

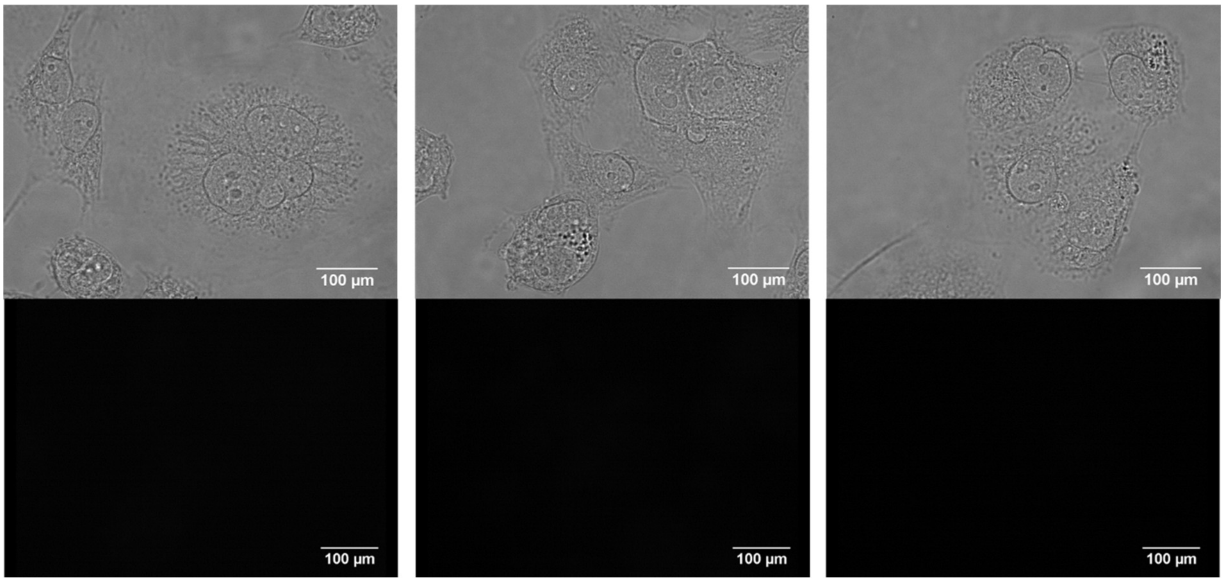

Figure S 39: Replicates of microscopy images (top) bright-field, (bottom) fluorescence at 488 nm of fixed MDA-MB-231 cells treated with LBB as vehicle control for 20 min, followed by irradiation for 15 min at 365 nm.

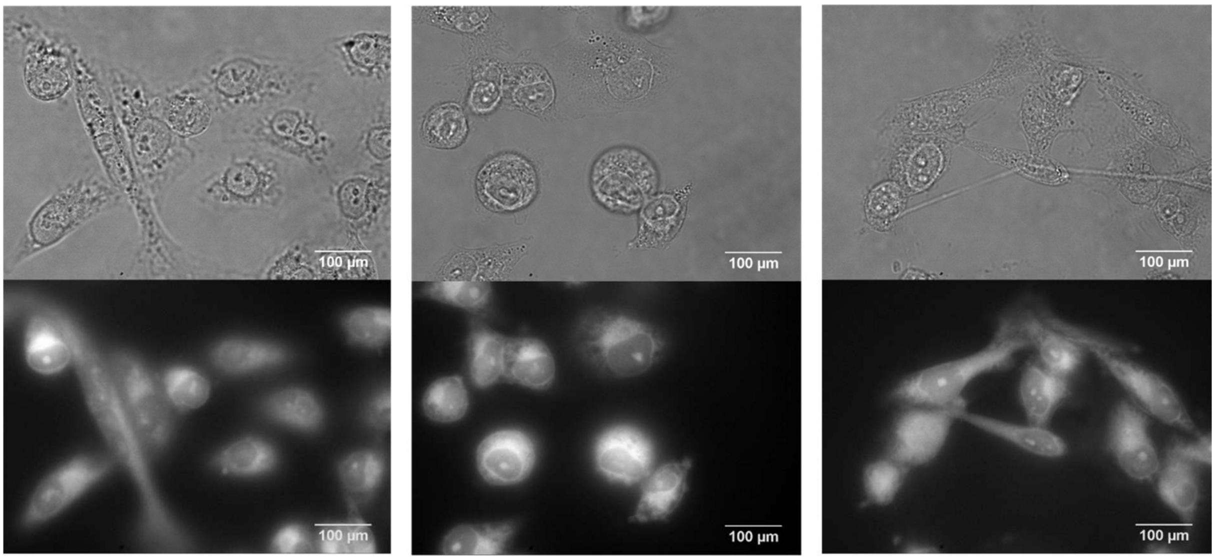

Figure S 40: Replicates of microscopy images (top) bright-field, (bottom) fluorescence at 488 nm of fixed MDA-MB-231 cells treated with AzCMan (10µM) for 20 min, followed by irradiation for 15 min at 365 nm.

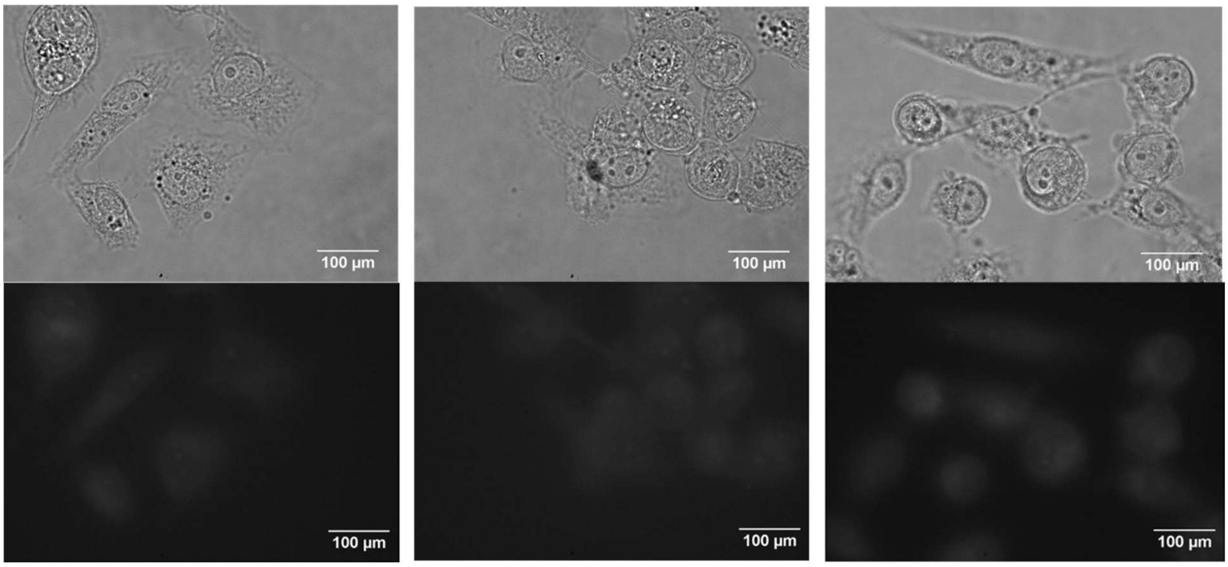

Figure S 41: Replicates of microscopy images (top) bright-field, (bottom) fluorescence at 488 nm of fixed MDA-MB-231 cells treated with pre-activated AzCMan (10µM) for 20 min, followed by irradiation for 15 min at 365 nm.

AzC activation by the UV laser of the microscope

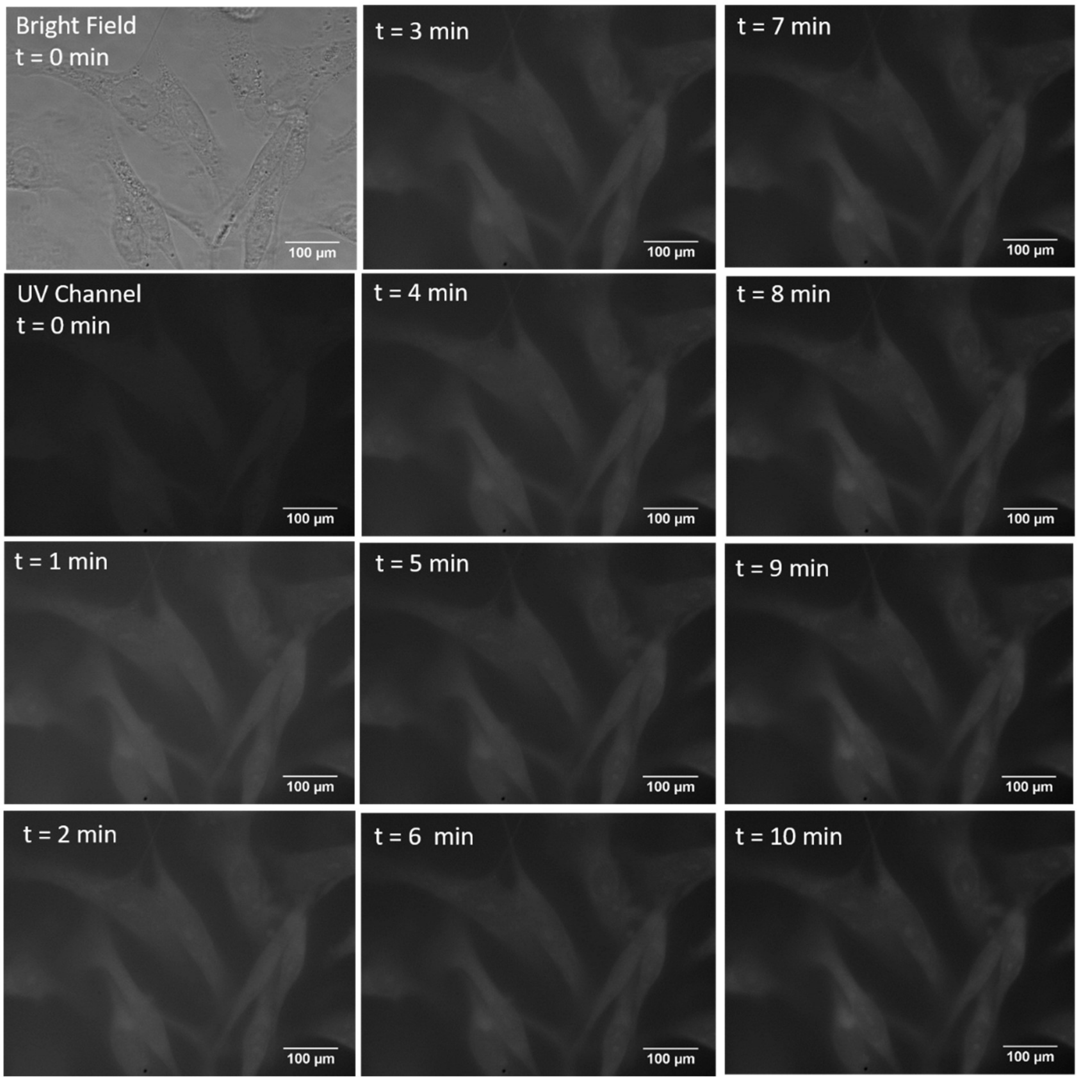

Figure S 42: Fluorescence microscopy images of photo-activation of the AzCMan probe using the UV laser of the microscope for a duration of 0 min to 10 min.
